# Understanding the development of tuberculous granulomas: insights into host protection and pathogenesis, a review in humans and animals

**DOI:** 10.3389/fimmu.2024.1427559

**Published:** 2024-12-09

**Authors:** Jiwon Lyu, Drew E. Narum, Susan L. Baldwin, Sasha E. Larsen, Xiyuan Bai, David E. Griffith, Véronique Dartois, Threnesan Naidoo, Adrie J. C. Steyn, Rhea N. Coler, Edward D. Chan

**Affiliations:** ^1^ Division of Pulmonary and Critical Medicine, Soon Chun Hyang University Cheonan Hospital, Seoul, Republic of Korea; ^2^ Department of Academic Affairs, National Jewish Health, Denver, CO, United States; ^3^ Center for Global Infectious Diseases, Seattle Children’s Research Institute, Seattle, WA, United States; ^4^ Division of Pulmonary Sciences and Critical Care Medicine, University of Colorado School of Medicine, Aurora, CO, United States; ^5^ Department of Medicine, National Jewish Health, Denver, CO, United States; ^6^ Center for Discovery and Innovation, Hackensack Meridian School of Medicine, Nutley, NJ, United States; ^7^ Departments of Forensic & Legal Medicine and Laboratory Medicine & Pathology, Faculty of Medicine & Health Sciences, Walter Sisulu University, Mthatha, South Africa; ^8^ Africa Health Research Institute, University of KwaZulu-Natal, Durban, South Africa; ^9^ Department of Microbiology and Centers for AIDS Research and Free Radical Biology, University of Alabama at Birmingham, Birmingham, AL, United States; ^10^ Department of Pediatrics, University of Washington School of Medicine, Seattle, WA, United States; ^11^ Department of Global Health, University of Washington, Seattle, WA, United States; ^12^ Department of Medicine, Rocky Mountain Regional Veterans Affairs Medical Center, Aurora, CO, United States

**Keywords:** tuberculosis, pathology, animal models, immunology, lung, mycobacteria, granuloma

## Abstract

Granulomas, organized aggregates of immune cells which form in response to *Mycobacterium tuberculosis* (*Mtb*), are characteristic but not exclusive of tuberculosis (TB). Despite existing investigations on TB granulomas, the determinants that differentiate host-protective granulomas from granulomas that contribute to TB pathogenesis are often disputed. Thus, the goal of this narrative review is to help clarify the existing literature on such determinants. We adopt the *a priori* view that TB granulomas are host-protective organelles and discuss the molecular and cellular determinants that induce protective granulomas and those that promote their failure. While reports about protective TB granulomas and their failure may initially seem contradictory, it is increasingly recognized that either deficiencies or excesses of the molecular and cellular components in TB granuloma formation may be detrimental to the host. More specifically, insufficient or excessive expression/representation of the following components have been reported to skew granulomas toward the less protective phenotype: *(i)* epithelioid macrophages; *(ii)* type 1 adaptive immune response; *(iii)* type 2 adaptive immune response; *(iv)* tumor necrosis factor; *(v)* interleukin-12; *(vi)* interleukin-17; *(vii)* matrix metalloproteinases; *(viii)* hypoxia in the TB granulomas; *(ix)* hypoxia inducible factor-1 alpha; *(x)* aerobic glycolysis; *(xi)* indoleamine 2,3-dioxygenase activity; *(xii)* heme oxygenase-1 activity; *(xiii)* immune checkpoint; *(xiv)* leukotriene A4 hydrolase activity; *(xv)* nuclear-factor-kappa B; and *(xvi)* transforming growth factor-beta. Rather, more precise and timely coordinated immune responses appear essential for eradication or containment of *Mtb* infection. Since there are several animal models of infection with *Mtb*, other species within the *Mtb* complex, and the surrogate *Mycobacterium marinum* – whether natural (cattle, elephants) or experimental (zebrafish, mouse, guinea pig, rabbit, mini pig, goat, non-human primate) infections – we also compared the TB granulomatous response and other pathologic lung lesions in various animals infected with one of these mycobacteria with that of human pulmonary TB. Identifying components that dictate the formation of host-protective granulomas and the circumstances that result in their failure can enhance our understanding of the macrocosm of human TB and facilitate the development of novel remedies – whether they be direct therapeutics or indirect interventions – to efficiently eliminate *Mtb* infection and prevent its pathologic sequelae.

## 1 Introduction

Granulomas are aggregates of immune cells that form in response to repetitive exposure to various stimuli that include infectious agents (*e.g.*, bacteria, fungi, protozoa, helminths, and viruses), non-infectious foreign bodies (*e.g.*, talc, starch, sutures), or an unknown inciting agent (*e.g.*, sarcoidosis, Crohn’s enteritis, granulomatosis with polyangiitis, polyarteritis nodosa, *etc.*) (1–3). While granulomas are typically regarded as a host-protective response against microbial agents, they may also lead to pathologic consequences, especially if the granulomas are unable to eradicate the microbes (2). In the context of tuberculosis (TB), granulomas are well documented in both humans and experimental animals (4).

The objective of this narrative review is to describe the factors that provide a host-protective granulomatous response with TB and the circumstances which cause granulomas fail. To help shed light on determinants of protective and non-protective TB granulomas, we also compared the TB granulomatous response and other pathologic lung lesions in various animals naturally or experimentally infected with *Mycobacterium tuberculosis* (*Mtb*), other *Mtb* complex species, or a mycobacterial surrogate with that of human pulmonary TB.

## 2 Cellular composition and functions of the TB granuloma

### 2.1 Overall structure of granulomas

**Figure 1:**
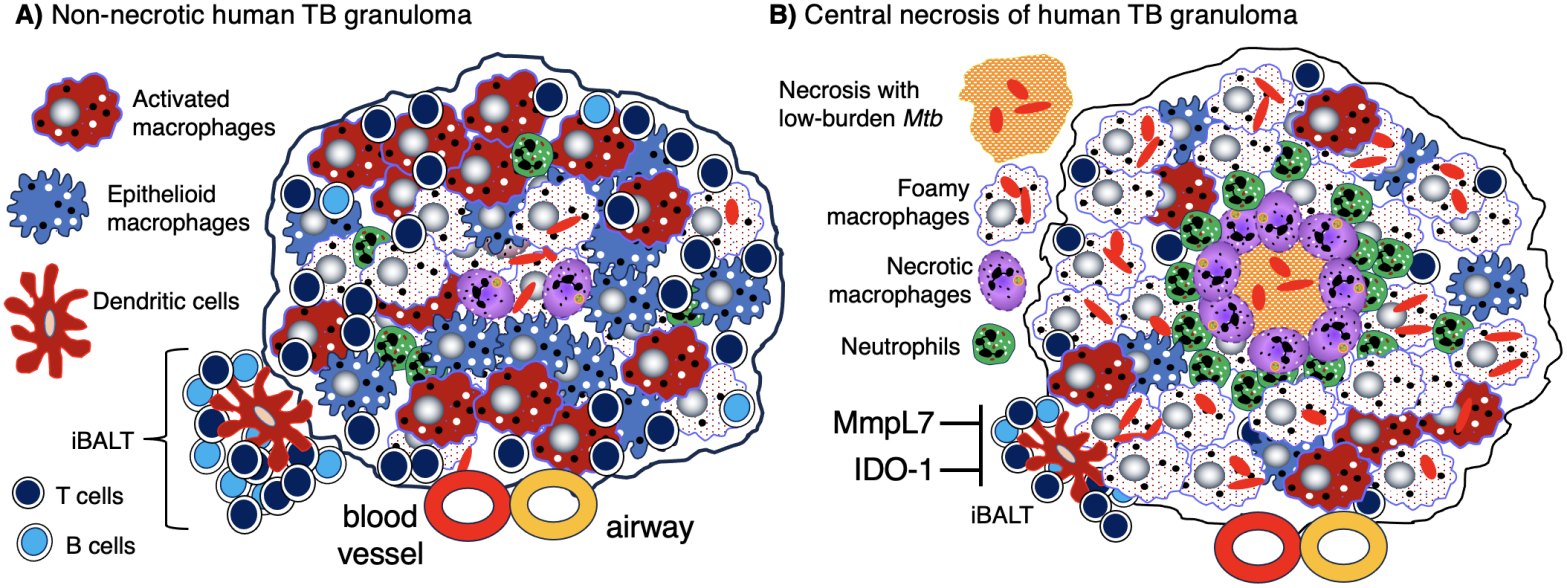
Human TB granulomas may be non-necrotic or necrotic. Several different fates of granulomas occur and such heterogeneity may present in the same individual. **(A)** A non-necrotic TB granuloma is characterized by the presence of epithelioid macrophages and activated macrophages as well as fewer foamy macrophages, necrotic macrophages, neutrophils, and more or less a rim of host-protective T cells and B cells along with relatively few bacilli. iBALT may be considered an “appendage” of granulomas typically comprised of dendritic cells, B cells, and T cells and may serve as a ready supply of immune cells for the neighboring granuloma. This granuloma is protective as shown by the relatively few *Mtb* (red-colored rod-shaped structures. **(B)** A granuloma with central necrosis characterized by fewer epithelioid and activated macrophages and more foamy macrophages, necrotic macrophages, and neutrophils along with an area of central necrosis. There are also fewer host-protective T cells and B cells. Due to the fewer number of host-protective innate and adaptive immune cells, there is overall a greater *Mtb* burden. However, in the central necrotic area of this human granuloma, there is active killing of *Mtb* resulting in fewer viable *Mtb* in this region. Higher levels of MmpL7 and IDO-1 inhibit iBALT formation. IDO-1, indoleamine 2,3-dioxygenase; iBALT, inducible bronchus-associated lymphoid tissue; MmpL7, Mycobacterial membrane protein Large 7; *Mtb*, *Mycobacterium tuberculosis*; TB, tuberculosis.

Granulomas are considered a key histopathologic feature of TB. Classically, the spatial organization of TB granulomas is comprised of a central region populated with macrophages (epithelioid, activated, and foamy macrophages) with an innermost zone that may become necrotic. Other myeloid cell types (dendritic cells, neutrophils, eosinophils, multinucleated giant cells, and/or mast cells) may be interspersed with the macrophages. Surrounding the macrophages are lymphocytes (T cells, B cells, natural killer cells, and innate lymphoid cells), forming a cuff in the outer zone of the granulomas (**Figures 1A, B**). Granulomas may also contain endothelial and epithelial cells from blood vessels and airways, respectively, as well as be encapsulated by collagen secreted by fibroblasts. However, granulomas may display great diversity with regards to certain immunological characteristics even within a single individual (5). In more protective granulomas, the concentric cell layers are gradually organized along with the presence of fibrosis, which suppresses necrosis, strengthens the lymphocytic rim, and leads to pathologic resolution (**Figure 1A**). These resolved granulomas can be identified radiologically and histologically by dystrophic calcification of the necrotic debris within these structures (6). In cases of active and progressive TB, granulomas have higher number of foamy macrophages and neutrophils as well as exhibit necrotic centers (**Figure 1B**).

**Figure 2:**
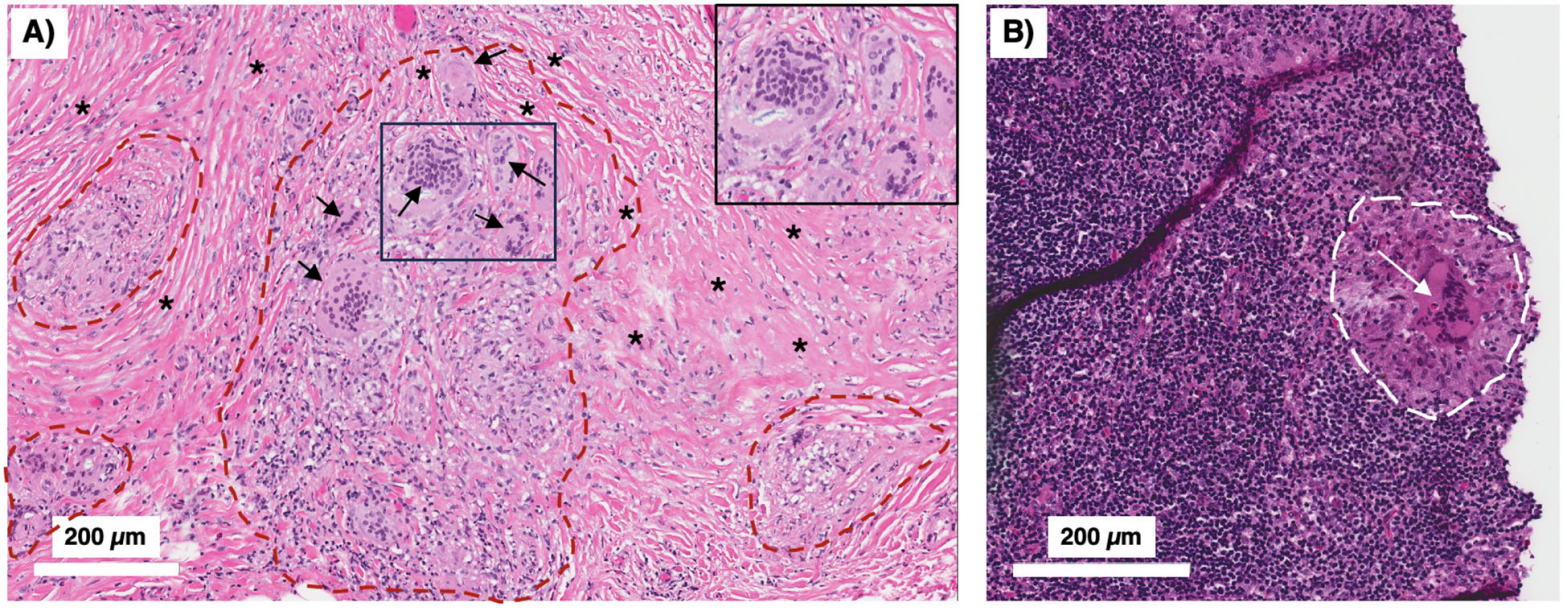
Peritoneal TB with non-necrotizing granulomas. A 51-year-old man from the Philippines presented with a two-year history of early satiety, intractable abdominal pain, and weight loss exceeding 60 pounds. Abdominal CT showed evidence of “peritoneal carcinomatosis.” **(A)** Peritoneal biopsy showed no evidence of malignancy. Instead, there were multiple non-necrotizing granulomas (demarcated by red dashed lines) with prominent Langhans-type multinucleated giant cells (arrows) and surrounding fibrosis (asterisks) (H&E). The boxed inset shows a magnified view of the smaller box demonstrating the multinucleated giant cells. **(B)** A peritoneal lymph node biopsy showed similar non-necrotizing granulomatous inflammation (white dashed line) with a conspicuous multinucleated giant cells (white arrow) (H&E). The acid-fast stain in both the peritoneal and lymph node biopsies were negative. With standard treatment for drug-susceptible TB, his symptoms abated and he regained his lost body weight. TB, tuberculosis.

Granulomas can be classified by several different morphological characteristics. One is whether cells within the central regions of granulomas are necrotic, which may be found in human TB, in natural TB in animals, and experimental TB in some laboratory animals. Caseation is the gross pathological term which alludes to the “cheese-like” material found when sufficient number of macrophages in the granuloma core undergo necrotic cell death. Caseating granulomas typically are consisted of necrotic, acellular core surrounded by epithelioid macrophages, and an outer lymphocytic cuff comprised of B and T cells (7). While the presence or absence of granuloma necrosis is often linked, respectively, to infectious (*e.g.*, TB) or non-infectious (*e.g.*, sarcoidosis) etiology for the granuloma, this dichotomy is not absolute. For example, necrosis may be seen in some non-infectious granulomas (2) and TB granulomas may not show any necrosis (**Figure 2**) (8).

Granulomas can show other morphological characteristics. Some granulomas may display fibrosis, characterized by collagen deposit either peripherally (those with a cuff of collagen surrounding them) or centrally (those with collagen throughout the entire granuloma) (9). Another subtype of granulomas is known as suppurative or neutrophil-rich granuloma. These granulomas arise when neutrophils infiltrate the center of the granuloma. Overall, high concentrations of neutrophils within *Mtb* granulomas have shown to be host destructive (enable spread of infection, higher levels of inflammation, and result in poorer host outcomes) (10). A calcified granuloma is typically regarded as the “final” stage of infection, and a sign of a successful host-immune response. The calcification (or mineralization) process typically begins within the caseous center (7, 11).

### 2.2 Selected immune cells that comprise granulomas

#### 2.2.1 Macrophages

Lung macrophages are ubiquitous in granulomas and along with dendritic cells are likely the first cell types in the nascent granuloma. These phagocytes engulf *Mtb* and orchestrate the influx of other cell types (2). After being engulfed, *Mtb* are killed when *Mtb*-containing phagosomes fuse with lysosomes. However, the bacilli may avoid this fate by inhibiting phagosome and autophagosome maturation (12–16). Ingested *Mtb* may also escape from arrested phagosomes by disrupting the phagosome membrane – *via* the release of *Mtb* pore-forming protein ESAT-6 (early secreted antigenic target-6) utilizing the ESX-1 (type VII) secretion system – and translocating into the cytoplasm (17, 18). In the cytosol, *Mtb* replicates, precipitating both necrosis of the phagocytes and release of bacilli extracellularly. Neighboring macrophages then phagocytose the released *Mtb* and the infection cycle repeats. During this cycle of suboptimal control of infection, the infected and subsequent necrotic macrophages release both inflammatory chemokines and mycobacterial cell wall components that induce influx of neutrophils and monocytes to the infection site, forming the early granuloma (19).

Infected macrophages and dendritic cells also migrate to the regional mediastinal lymph nodes to help differentiate and recruit antigen-specific T cells (*e.g.*, T_H_1 and T_H_17), which then migrate to the infection site, activate monocytes and macrophages, and participate in the formation of evolving granulomas (15, 16, 19–21). This delayed influx of recruited T cells to the site of the infection may allow *Mtb* to persist if the initial macrophages encountered are of the more “permissive” phenotype (22). However, with sufficient recruitment of *Mtb*-specific and activated T cells to the granulomas, containment or eradication of *Mtb* within the granulomas is possible. Macrophages in evolving granulomas may be comprised of various phenotypes, including “classically activated”/inflammatory (M1) macrophages and various subsets of “alternatively activated” (M2) macrophages as well as other morphologically distinct macrophages (epithelioid macrophages, foamy macrophages) that likely have overlapping features with M1 or M2 macrophages, and multinucleated Langhans giant cells (23, 24).

M1 macrophages are activated in response to T_H_1 T-cell signals (interferon-gamma [IFNγ] and tumor necrosis factor [TNF]) as well as lipopolysaccharide (LPS) (7). Once activated, M1 macrophages secrete pro-inflammatory cytokines (TNF, interleukin-1 [IL-1], IL-6) and inducible nitric oxide synthase (iNOS). Whereas the M1 response is important in TB control, an excessive response can lead to chronic inflammation as well as inflammatory diseases (25). On the other hand, M2 macrophages are known to inhibit the inflammatory response, playing an important role in wound healing and tissue repair. These macrophages are activated by T_H_2 T-cell cytokines (IL-4 and IL-13). In response, they produce anti-inflammatory cytokines (IL-10, transforming growth factor-beta [TGFβ]), IL-6, and arginase (7, 25). However, this binary categorization is likely an oversimplification to fully describe the function of these subtypes, especially M2 macrophages which are divided into at least three sub-phenotypes (26, 27). We expound below two distinct macrophage phenotypes given their preponderance in granulomas: “epithelioid macrophages” and “foamy macrophages” (2, 28).

##### 2.2.1.1 Epithelioid macrophages

Epithelioid macrophages are so named due to their cellular characteristics that resemble epithelial cells: spread morphology, elongated nuclei, high cytoplasm-to-nucleus ratio, and contact interaction with neighboring macrophages. Epithelioid macrophages are also less phagocytic and more secretive than activated macrophages (29, 30). Additional characteristics of epithelioid macrophages are discussed in **Table 1**.

**Table 1:** Epithelioid and foamy macrophages in TB granulomas.

Cell Type	Location in granuloma	Role in mycobacterial infection	Stimulus that drives production
**Epithelioid macrophages**	Surrounding the necrotic center and interspersed in the cellular rim (34)	Depletion leads to increased intracellular bacillary numbers (35) whereas excessive formation “walls off” infection site from other immune cells and antibiotics (36, 37)	IL-4 activation of STAT6, which mediates expression of E-cadherin, adhesion molecule that is crucial in transforming cytoskeletal structure of epithelioid macrophages through production of adherens junctions, desmosomes, & tight junctions (11, 35, 37)
**Foamy macrophages**	Surrounding the necrotic center; rarely found in non-necrotic granulomas (32)	Contributes to caseous necrosis (by discharging intracellular lipids & *Mtb*) & have less bactericidal/phagocytic compared to other macrophages due to fat accumulation, creating long-term persistence of *Mtb* in these cells (31, 38); fat accumulation occurs due to ESX-1 driven metabolic shift from glycolysis to ketogenic pathway synthesis, inducing expression of G-protein coupled receptor GPR109A which inhibits lipolysis (39) (**Figure 3**); fat accumulation decreases autophagy due to lower expression of lactate (byproduct of glycolysis), which normally increases autophagy by *lactylation* of class III phosphatidylinositol 3 kinase) (39–42); reduced autophagy further attenuates glycolysis and ketogenic pathway (*i.e.*, oxidative phosphorylation), the latter via autophagy mediated metabolism of triglycerides into fatty acids, which are then broken down into acetyl Co A in the mitochondria for ATP production (43–45).	*Mtb* derived oxygenated mycolic acids bind to host lipid sensing nuclear receptors PPARγ and TR4, inducing production of host derived LDL receptors (scavenger receptor A and CD36) (31, 38, 46, 47); these receptors bind and mediate influx of host derived LDL intracellularly (**Figure 3**); ESX-1 drives foamy macrophage formation in *M. marinum*-zebrafish model (48); IL-10 also shown to induce differentiation to foamy macrophages (49)

ESX-1, ESAT-6 protein family secretion system (ESAT-6, 6 KDa early secretory antigenic target); IL-4, interleukin-4; LDL, low density lipoprotein; PPARγ, peroxisome proliferator-gamma; TR4, testicular receiver 4.

##### 2.2.1.2 Foamy macrophages

**Figure 3:**
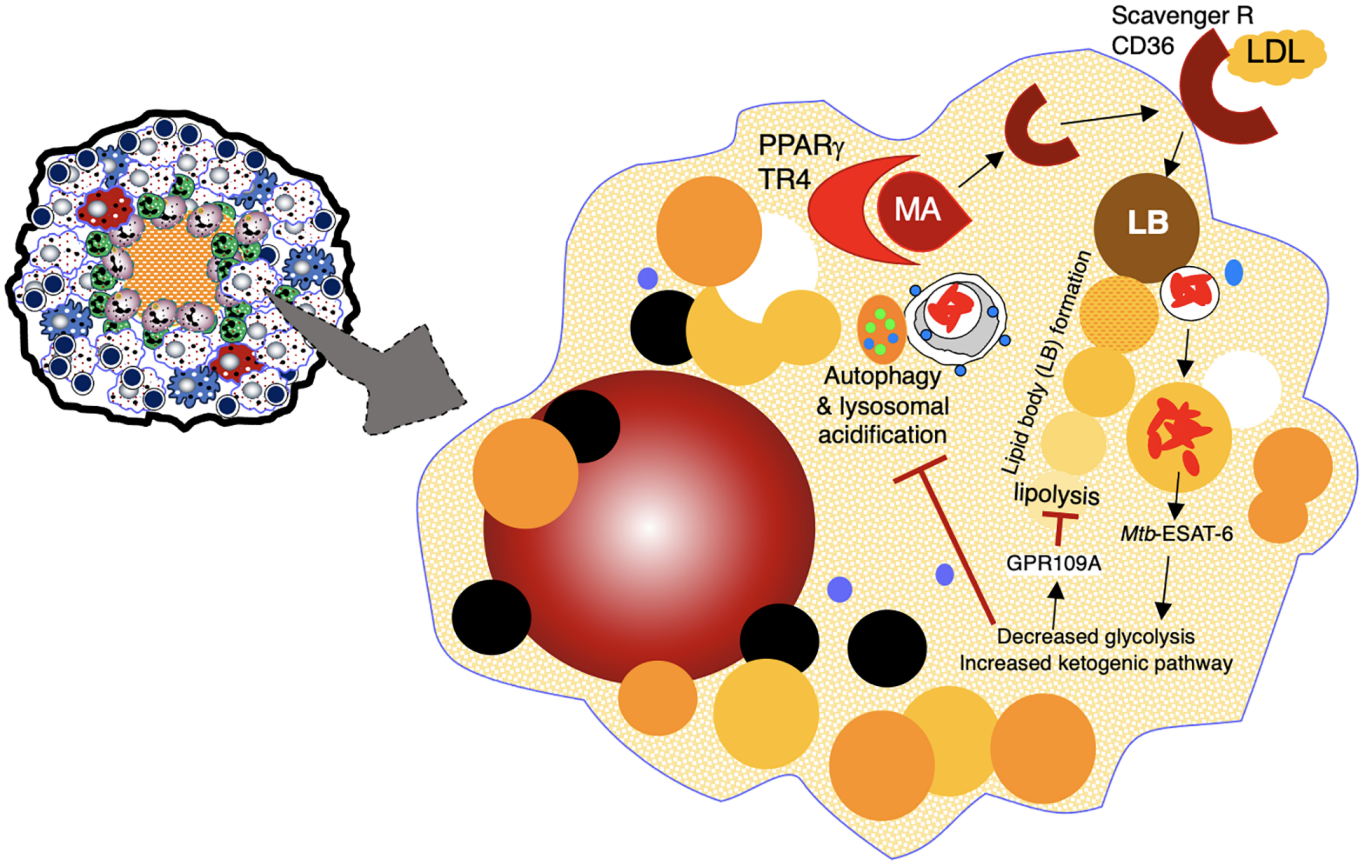
Formation of foamy macrophages and their role in TB pathogenesis. Foamy macrophages play a key role in the formation of caseous necrosis. They are formed when macrophages acquire cholesterol-containing LDL particles into lipid bodies. See text for discussion. ESAT-6, 6 kDa early secretory antigenic target; GPR109A, a specific G-protein coupled receptor; LB, lipid bodies; LDL, low density lipoprotein; MA, mycolic acids; PPARγ, peroxisome proliferator-gamma; TR4, testicular receiver 4.

Macrophages in some TB granulomas appear foamy due to the accumulation of various intracellular lipids (cholesterol, triglycerides, and phospholipids) (31). In foamy macrophages, bacilli are observed in proximity to lipid droplets, posited to be a nutrient source for *Mtb* (29). Foamy macrophages are typically found around the edge of the necrotic core of caseating granulomas and rarely found in non-necrotic granulomas (**Figures 1**, **3**) (32). In human autopsy cases of TB, foamy DEC-205^+^ dendritic cells were more common in lipoid pneumonia and cavitary lesions than in granulomas (33). Additional characteristics of foamy macrophages are discussed in **Table 1**.

##### 2.2.1.3 Apoptotic vs. necrotic cell death of phagocytes in granulomas

Death of *Mtb*-containing phagocytes may either be a mechanism of restricting *Mtb* replication or spreading the infection. Apoptosis of *Mtb*-infected cells is a mechanism by which phagocytosed intracellular bacilli are killed (50–58). In addition, macrophage phagocytosis of apoptotic bodies – a process known as efferocytosis – can further enhance the killing of *Mtb* contained within the apoptotic bodies (59). Thus, apoptosis is a host protective mechanism to not only dispose of phagocytosed *Mtb* but to limit excessive inflammation associated with necrosis (58). In contrast, in the zebrafish-*M. marinum* model, phagocytosis of apoptotic macrophages infected with *M. marinum* promoted the spread of mycobacteria (60).

In contrast, cell necrosis permits survival and release of *Mtb* to infect neighboring cells (58, 61–63). Necrosis may be dictated, in part, by the *Mtb* strain and thus an immune evasive virulence phenomenon. Divangahi et al. (64) demonstrated that the more virulent *Mtb* H37Rv, in contrast to the less virulent *Mtb* H37Ra, induced the lipoxin A4 (LXA_4_) production, which then blocks both synthesis of prostaglandin E_2_ (PGE_2_) and activation of synaptotagmin 7. Since PGE_2_ prevents necrosis of *Mtb*-infected macrophages (61, 64–66) and synaptotagmin 7 (a calcium ion sensor) is involved with lysosome-mediated repair mechanism (64), LXA_4_ induced by the more virulent *Mtb* prevents repair of the injured plasma membrane caused by *Mtb*, resulting in cellular necrosis. Another mechanism by which *Mtb* induces necrosis is *via* activation of the cytosolic receptor-interacting protein kinase 3 (RIPK-3) pathway, preventing apoptosis of *Mtb*-infected macrophages through Bcl-xL and promoting reactive oxygen species (ROS)-mediated cell necrosis (67).

#### 2.2.2 Neutrophils

Neutrophils can phagocytose *Mtb* as well as release neutrophil extracellular traps (NETs), which are comprised of chromatin (DNA) decorated with neutrophil-derived proteins (elastase, histones) that entrap the extracellular bacilli. NETs have antimicrobial activity and the NET-induced inflammatory response may promote migration of activated dendritic cells to the draining lymph nodes to activate naïve T cells; however, excessive NET-associated inflammation also causes tissue injury (68, 69). In addition, type 1 interferon-induced NET formation was found to promote *Mtb* growth and disease severity in mice genetically susceptible to TB (65). Neutrophils may also be present at the interface between the necrotic center and epithelioid macrophages as well be a source of immunoregulatory cytokines TNF and IL-10 (70). In C3HeB/FeJ mice, a fulminant necrotizing alveolitis with uncontrolled neutrophilic response in the lungs is associated with greater *Mtb* burden and mortality (34). Neutrophils may also provide a niche for mycobacterial growth, and this ability was associated with increased mitochondrial metabolic activity (71, 72).

#### 2.2.3 Lymphocytes

##### 2.2.3.1 CD4^+^ T effector cells

CD4^+^IFNγ^+^ T cells are critical in the formation of granulomas with early containment of *Mtb* (73–75). However, excessive amounts of CD4^+^IFNγ^+^ T cells, in conjunction with increased TNF, can also cause macrophage necrosis and poorer control of *Mtb* (76, 77). Similarly, either excessive or insufficient CD4^+^IL-13^+^ T cells (37, 78) impairs host control of *Mtb* (79–81). However, BALB/c mice with genetic disruption of IL-4 were more proficient in controlling *Mtb* (82), indicating that attenuating T_H_2 (IL-4, IL-13) immunity in more immunocompromised mice is host-protective. TGFβ produced by macrophages and T regulatory cells (Tregs) predisposes to *Mtb* in a computer simulated TB granuloma model, in experimental animals, and in humans (83–85). However, TGFβ combined with IL-23 promotes the expansion of T_H_17 cells, which are important in the control of *Mtb* during earlier stages of infection (86). IL-17 secreted by T_H_17 cells has other host-protective functions (limiting excessive granuloma hypoxia, inducing iBALT formation, recruiting CD4^+^IFNγ^+^ T cells) (87–90) but also host-deleterious properties (inducing excessive influx of neutrophils that may limit the formation of protective granulomas) (91, 92). Serum IL-9 (produced by T_H_9 cells) was found to be significantly higher in pulmonary TB subjects than individuals with sarcoidosis, a granulomatous disorder of unknown etiology (93).

##### 2.2.3.2 CD8^+^ T effector cells

CD8^+^ T cells, through their cytotoxic response, help limit bacterial replication in the later stage of disease (6, 94). Following aerosol *Mtb* infection of mice, CD8^+^ cells were not considered to play a major protective role (95) whereas with high intravenous inoculum, they were more essential for protection (96–98). CD8^+^ T-cells are able to recognize and induce apoptosis of infected macrophages (99). In mice, CD8^+^ T cells were found to be important in maintaining stable latent phase of infection, in part through production of IFNγ (100). However, increased TGFβ and IL-10 expression and reduced granzyme B production by CD8^+^ T cells are associated with poorer control in patients with active TB (101).

##### 2.2.3.3 T regulatory cells

Tregs are CD4^+^ T cells (or less frequently CD8^+^ marker) characterized by the phenotypic markers Foxp3^+^CD25^hi^CD127^dim/–^. Tregs develop from naïve T cells in the presence of TGFβ and dampen the function of M1 macrophages and T effector cells through expression of immunosuppressive cytokines TGFβ, IL-10, and IL-35 (102–104). Tregs were more commonly present in cavities than in the lipoid pneumonia or granulomas of human post-primary TB in the lungs (33) Within granuloma-infiltrating T cells of both *Mtb*-infected mice and rhesus macaques, TGFβ signaling antagonized activity of IFNγ-producing CD4^+^ T cells and impaired control of *Mtb* (83).

##### 2.2.3.4 B cells

B cells also play an important role in the granulomatous and iBALT response against *Mtb* (105, 106). B cells found in granulomas produce the chemokine CXCL13, which recruits specific T cells to the infection site, promoting follicle-like structure formation within granulomas (107). B cells also function as antigen-presenting cells within granulomas, enhancing proliferation of a host-protective T cells and plasma cells against *Mtb* infection (108). Studies in mice have shown that B cells decrease neutrophil motility (109–111).

Three distinct populations of B cells exist: B effector 1 cells (Be1), B effector 2 cells (Be2), and regulatory IL-10 producing B cells (B10). Be1 cells are produced when naïve B cells are primed by T_H_1 cytokines. These cells then produce IFNγ, IL-12, TNF, IL-10, and IL-6. On the other hand, Be2 cells are differentiated in the presence of T_H_2 cytokines, and, in turn, are producers of IL-2, lymphotoxin, IL-4, IL-13, IL-10 and IL-6 (105, 112, 113). Once produced, Be1 and Be2 cells are able to influence the differentiation of naïve CD4^+^ T cells into T_H_1 or T_H_2, respectively (112). Regulatory B cells are known to downregulate the T cell response through production of IL-10 or TGFβ (112, 113). Thus, whereas effector B cells induce the inflammatory T cell response, regulatory B cells downregulate this pathway. Due to the crucial role that CD4^+^ T cells play in the formation of TB granulomas, the ability of B cells to induce or block this pathway illustrates their importance in granuloma formation. Indeed, clusters of B cells are found in human TB granulomas (114).

### 2.3 Inducible bronchus-associated lymphoid tissue, an accessory organoid to granulomas

Tertiary lymphoid organs (TLOs) are organized lymphoid structures that form in non-lymphoid tissues in response to injury, inflammation, or chronic infection (115). These structures have been shown to surround granulomas during mycobacterial infection (116, 117). While TLOs and granulomas are generally regarded as discrete structures, they have been shown to share similar biological processes (118). One such TLO that is associated with granuloma formation is inducible bronchus-associated lymphoid tissue (iBALT). iBALT are comprised of B cells, T cells, and antigen-presenting cells (*e.g.*, dendritic cells) and are juxtaposed to granulomas (**Figures 1A, B**) (119–121). The immune cell inflammation that accompanies *Mtb* infection: *(i)* induces “stromal lymphoid tissue organizer cells” (fibroblasts, myofibroblasts, and endothelial cells) to transform into follicular dendritic cells; *(ii)* triggers them to produce chemokines (CXCL13, CXCL12, CCL21, CCL19), which recruit and organize T cells, B cells, and dendritic cells into iBALT; and *(iii)* supports high endothelial venule formation to allow lymphocyte trafficking into the infection site (122). Interleukin-17 (IL-17), IL-23, and group 3 innate lymphoid cells (ILC3s) also promote the differentiation and activation of lymphoid cells that participate in iBALT formation (89, 90, 123–125).

iBALT has been described with pulmonary TB in humans (75, 121, 126), non-human primates (NHP) (75, 121, 126), rabbits (127), guinea pigs (127), and mice (75, 125). iBALTs are considered to be protective against *Mtb* for several reasons: *(i)* they can serve as a site for local antibody production (128, 129); *(ii)* they can render support to neighboring granulomas as a ready supply of additional immune cells (119, 130); *(iii)* the presence of iBALT can be associated with the maintenance of latency and containment of infection, whereas its absence is associated with active disease (75, 121). However, in the context of sustained and unresolved TB, iBALT may become pathogenic and contribute to lung injury, especially with sustained IL-17 expression and neutrophil recruitment (119).

Certain molecules can have an impact on iBALT formation. For example, NHP infected with *Mtb* that lacked MmpL7 (Mycobacterial membrane protein Large 7) showed an increase in granulomas containing iBALT, suggesting that *Mtb*-derived MmpL7 prevents the formation of host-protective iBALT (**Figure 1B**) (131). Another study found in both *in vitro* (macrophage) and *in vivo* (murine and NHP) *Mtb* models that inhibition of indoleamine 2,3-dioxygenase (IDO-1) activity resulted in a greater host protective response as well as increased iBALT, indicating that IDO-1 also inhibits iBALT formation (**Figure 1B**) (132).

## 3 The granuloma paradox

### 3.1 Granulomas may be viewed from different perspectives

One overarching paradox of TB is that despite being the most lethal infectious disease globally, most who are infected with *Mtb* do not ever develop disease, consistent with pathologic studies that most human TB lesions are regressive (133, 134). Because humans and *Mtb* have co-existed for a long time, it is likely that the genetics of both humans and *Mtb* are important in determining who are at risk for developing latent infection or active TB disease (135, 136). The battle between *Mtb* and host immunity may be viewed from either the perspective of the bacilli or the host. When the host is unable to completely eradicate the *Mtb* upon initial infection – due to immune evasive strategies of *Mtb* such as arrest of phagosome maturation (137) – the next best recourse is to contain the infection through the formation of granulomas. While granulomas are generally regarded as a host-protective response by controlling the *Mtb* infection, they also provide a niche that allows *Mtb* survival by limiting the trafficking of immune cells (4). Some newly infected macrophages may also escape from the original granuloma to form a secondary granuloma, albeit this was observed in the *M. marinum*-zebrafish model (60).

Drawing from epidemiological evidence indicating that the majority of individuals with latent TB infection do not progress to active TB but remain at risk, our discussion framework centers on the host-protective nature of TB granulomas. We explore the multifaceted circumstances leading to their occasional failure in protecting the host. There are many factors that determine whether TB granulomas are protective or harmful. One plausible explanation for variability in the protective efficacy of granulomas lies in the existence of different granuloma phenotypes – a heterogenous spectrum of lesions – within the same individual. These distinct granuloma types emerge due to a complex interplay between the host immune response and *Mtb* components, reflecting varying endotypes (138). However, a glaring limitation emerges when assigning a specific granuloma phenotype as detrimental to the host: the challenge of discerning whether a particular granuloma phenotype fails to protect or if it arises as a consequence of such failure or an attempt to correct the failure. In essence, our comprehension of granulomas is significantly hindered by our inability to observe the temporal evolution of individual granulomas in the context of live bacilli burden. Nevertheless, we have organized below the descriptions of granulomas in the context of host-protection or not.

### 3.2 Evidence that granulomas serve host-protective function

#### 3.2.1 Epidemiological and clinical observations

The notion that TB granulomas are host-protective is evinced indirectly from the observation that most individuals infected with *Mtb* – identified through positive tuberculin skin tests or IFNγ release assays – do not progress to active TB and commonly exhibit calcified granulomas. Indeed, global data show a much greater preponderance of individuals infected with *Mtb* without disease (by ~200-fold) than those with active TB (139).

A stronger piece of clinical evidence that granulomas serve a host-protective function is that individuals with severe immunodeficiency (such as those with AIDS or immune suppression by pharmacologic TNF blocking agents) are highly susceptible to *Mtb* and have poorly-formed or absent granulomas (140–145). On a cellular level, TNF is necessary for macrophage-mediated control of mycobacterial infection (146). The requirement of TNF in forming a host-protective TB granuloma is evinced by the finding that mice knocked out for TNF or neutralized of TNF function were unable to form granulomas in response to *Mtb* infection and showed greater *Mtb* burden (147). One novel function of TNF in granuloma formation is the induction of a T cell receptor (TCR) αβ-based recombinatorial immune receptor in subpopulations of monocytes/macrophages (148). As macrophage-TCRαβ was found to induce the release of CCL2 (monocyte chemoattractant protein 1), which recruits monocytes, dendritic cells, and memory T cells, deletion of this variable macrophage-TCRαβ or TNF results in structurally compromised TB granulomas (148). Yet, there are case reports of caseating granulomas (often a sign of high *Mtb* burden) found in active TB in the presence of TNF blocking agents, indicating that granulomas that form despite TNF deficiency are not protective (149, 150). One possible scenario for this finding is that latent TB granulomas previously existed but with the introduction of an anti-TNF agent, there was proliferation of dormant *Mtb*, causing disruption of the intact, protective granuloma with secondary necrosis. An alternative explanation is that during treatment with an anti-TNF agent, a new *Mtb* infection occurred (plausible in TB endemic countries), resulting in inadequate control of the initial infection and the recruitment of other cell types such as neutrophils, resulting in caseating necrosis of the poorly protective granulomas. The observation that most of the *Mtb* are extracellular within necrotic granulomas, with lesser number located in the macrophage-rich region surrounding the core would support either scenario (151).

#### 3.2.2 HIF-1α, a response to granuloma hypoxia, is largely host-protective

In non-necrotic, cellular granulomas, *Mtb* exists mainly intracellularly in the infected macrophage population within the core of the intact granuloma (11, 151, 152). One likely factor that allows granulomas to remain intact and non-necrotic is the prevention of a hypoxic environment within granulomas. More specifically, if cellular hypoxia occurs, one thwarting response is through increased production of hypoxia inducible factor-1 alpha (HIF-1α) by innate immune cells (macrophages, dendritic cells, and neutrophils) and lymphocytes (153). HIF-1α, which combines with constitutive HIF-1β to form HIF1, increases angiogenesis to the granuloma *via* induction of vascular endothelial growth factor, which allows oxygen and nutrient transport to the cells within granulomas (154). HIF-1α also aids cell survival in the hypoxic environment by augmenting glycolysis to generate quickly available ATP (155–157).

Several studies have shown that inducible HIF-1α is host-protective against *Mtb* infection. HIF-1α enhances the *Mtb*-killing abilities of both phagocytes and monocytes by several effector mechanisms including apoptosis of *Mtb*-infected phagocytes and enhanced production of cellular anti-microbial elements (nitric oxide, granule proteases, and anti-microbial peptides) (154, 158–160). Live *Mtb* alone has been shown to induce HIF-1α (154, 161) but this effect was especially seen in conjunction with IFNγ (162). In turn, HIF-1α augments IFNγ-induced genes that play important roles in effector functions against *Mtb* such as inflammatory cytokines and chemokines (IL-1α, IL-1β, and IL-6) (154, 162, 163). Hypoxia-induced HIF-1α also induces expression of CXCR4 – the receptor for the lymphocyte chemokine (and pro-angiogenic) CXCL12; thus, HIF-1α maintains both the influx of potentially host-protective immune cells to granulomas and the viability of these cells (164, 165). These findings are supported by studies of mice with conditional genetic disruption of HIF-1α in myeloid cells wherein with *Mycobacterium avium* infection, there was greater granuloma necrosis (perhaps due in part to impaired neovascularization of the granulomas) and impaired clearance of the mycobacteria (166). In the *M. marinum*-zebrafish model, stabilization of HIF-1α at the early stages of infection enhanced the levels of nitric oxide and reduced mycobacterial burden (167). Furthermore, suppression of granuloma-associated angiogenesis – which would be expected to cause hypoxia and induce the expression of HIF-1α – decreased *M. marinum* burden (168). While the ability of HIF-1α to enhance host immune cells against mycobacteria may be mediated by NFκB activation (169), HIF-1α may also attenuate an excessive NFκB response to prevent injurious inflammation (170).

### 3.3 Evidence that granulomas serve to propagate *Mtb*

#### 3.3.1 Is bacillary patience a survival factor for Mtb? A teleological perspective

The first observation that certain granulomas – depending on the model used – benefit *Mtb* growth is that *Mtb* has adapted to remain quiescent for long periods with the opportunity to propagate at later times. Unlike pathogens that do not develop latency in the host and evade host immunity by antigenic variation, *Mtb* is capable of surviving (in a latent form), paradoxically, *because* of its recognition by host immune cells. The notion that *Mtb* has exploited recognition by the host as a survival mechanism is supported by the extensive diversity of *Mtb* antigens and epitopes that are recognized by CD4^+^ and CD8^+^ T cells of both active TB subjects and healthy controls (171–173). Hence, a potential interpretation is that T-cell recruitment to the site of infection, although initially a host-protective mechanism, could inadvertently aid *Mtb* by facilitating the establishment of a latent infection within an organized granuloma. This arrangement extends the bacilli’s survival, enabling them to lay dormant until conditions conducive to their reactivation arise, such as during immune senescence, thereby facilitating their dissemination to other individuals (6, 174, 175). Therefore, a teleological perspective suggests that *Mtb*, by exhibiting remarkable “patience” in remaining quiescent after encountering host immune cells within granulomas, ensures a higher probability of long-term survival for its species.

#### 3.3.2 The co-evolution of Mtb with its host to survive within granulomas

The second observation that granulomas benefit *Mtb* is that the bacilli have developed survival mechanisms within the host phagocytes. A denouement of such survival capability is that the bacilli can spread between phagocytes within either quiescent or disruptive stages of granulomas. Mechanisms for this intracellular survival and spread to other cells are *Mtb*-mediated rupture of the phagosome membrane to escape into the cytoplasm, evading phagosome-lysosome fusion (17) as well as apoptosis-mediated cell-to-cell spread (60, 176). This intracellular movement of mycobacteria is likely an immune evasive mechanism because pathogenic mycobacteria are more capable of translocating from the intra-phagosome compartment to the cytosol *via* pore-inducing mycobacterial ESAT-6 protein than non-pathogenic mycobacteria (discussed in Section II.2.1 above) (17, 18). A connection between this intracellular immune evasive mechanism and the earlier-discussed *Mtb* latency survival strategy within organized granulomas emerges. ESAT-6, known to contain multiple T-cell epitopes (both CD4 and CD8), plays a pivotal role (177). Interestingly, just before *Mtb* burden plateaued post-infection in mice, the mRNA levels of both ESAT-6 and Ag85B decreased by 10-fold (178). This decline, reflecting little turnover of *Mtb* during the stationary phase, allows for *Mtb* to be still recognized by T cells but prevented its complete eradication by the immune system.

ESAT-6 secretion systems (ESX), particularly ESX-1 and ESX-5, have been shown to be virulence factors during mycobacterial infection. The importance of ESX-1 in mycobacterial pathogenesis is evinced by the finding that *Mtb* strains lacking ESX-1 have decreased pathogenesis and intracellular replication compared to wild-type strains (179). In humans, ESX-1 induction of the chemokine fractalkine (CX3CL1) mediates the recruitment of monocytes to the expanding granuloma (180). In contrast, the ESX-1 of *M. marinum* induced expression of matrix metalloproteinase 9 (MMP-9), which facilitates influx of uninfected macrophages, expanding the granulomatous lesions that may contain viable mycobacteria (181, 182). ESX-5 of *M. marinum* has been shown to inhibit induction of proinflammatory cytokines (IL-12p40, TNF, IL-6) (183) as well as induce both inflammasome/IL-1β activation and caspase-independent cell death (184).

*Mtb* also secretes lipids that affect the host immune response and caseous granuloma formation. These include mycolic acids, specifically trehalose 6,6’-dimycolate (TDM, aka cord factor), a toxic lipid that induces epithelioid macrophage transformation, pro-inflammatory cytokine production, and necrosis of the granulomas (185–187). Experimentally, the largest oil droplets of oil-water emulsions containing TDM were the most granuloma-genic (188). In contrast, de-lipidated BCG – where TDM was stripped from the outer cell wall – induced more of an acute (neutrophilic-like) infiltration and reduced delayed hypersensitivity (granulomatous) response along with more rapid clearance of de-lipidated BCG in mice, indicating that TDM is both a virulence factor and responsible for granuloma formation (189). The programming *Mtb* into a dormancy state by the dormancy survival regulon (DosR) is induced by carbon monoxide and hydrogen sulfide as well as a local environment that is hypoxic, acidic, and contains high nitric oxide levels (190–195). This induced dormancy of *Mtb* results in reduced metabolic activities of the bacilli, but they are also able to dysregulate host-lipid synthesis resulting in the production of lipid bodies (39). This process transforms macrophages into the foamy phenotype capable of not only sustaining the dormant *Mtb* but contributing to the caseous necrosis when granulomas breakdown (31).

*Mtb* has the capacity to import and catabolize host-derived cholesterol (an integral part of cellular membranes) – *via* proteins encoded by the mycobacterial gene cluster *mce4* and by the *igr* locus, respectively – and use it as a carbon and energy source to survive for prolonged periods in caseous granulomas (196, 197). The catabolism of cholesterol in host-cells requires a shift from aerobic to anaerobic metabolism within the hypoxic and necrotic granuloma core which contributes to the dormant state of *Mtb*. In this state, *Mtb* remodels the cell wall and produces intracellular triglycerides (comprised of three long-chain fatty acids esterified to glycerol). β-oxidation of even-chain fatty acids produces acetyl-Co-A, which can be used for either synthesizing triglycerides (a lipid relevant for dormancy) or fueling the tricarboxylic acid (TCA) cycle (for ATP production) (198). Odd chain fatty acids undergo β-oxidation to produce the 3-carbon propionyl-CoA, which can be further metabolized to products that enter the TCA cycle or produce methyl-branched lipids to synthesize the mycobacterial cell wall (198). The mycobacterial enzyme isocitrate lyase (Icl) serves two functions for *Mtb*: *(i)* it is essential for conversion of even-chain fatty acids under nutrient limitation; *i.e.*, Icl metabolizes isocitrate (6C) to glyoxylate (2C) and succinate (4C) with subsequent combining of glyoxylate with acetyl-Co-A (2C, a product of β-oxidation of fatty acids) to form malate (4C), which enters and fuels the tricarboxylic acid cycle and *(ii)* by fueling the TCA cycle, it activates the events that occur downstream of the initial steps in the metabolism of the toxic propionyl-CoA. In addition, the gene that encodes the lipid transporter also plays an important role in *Mtb* growth (6, 199).

#### 3.3.3 What causes necrosis and caseation of TB granulomas?

The root cause of the necrotic cell death and subsequent caseation in TB granulomas is not precisely known. Within TB granulomas, macrophage necrosis occurs by various and not necessarily mutually exclusive mechanisms. The first mechanism is dysregulation of the pro-inflammatory cytokine TNF. On the one hand, either deficiency or antagonism of TNF impairs macrophage control of *Mtb* (and of *M. marinum*), increases mycobacterial burden in macrophages, and results in necrotic macrophage death with subsequent tissue spread of *Mtb* (4, 76, 146, 200). This TNF deficiency may also occur endogenously in certain individuals due to decreased leukotriene A4 hydrolase (LTA4H) activity (CC genotype of *LTA4H* gene), resulting in a decrease ratio of the pro-inflammatory leukotriene B4 (LTB_4_) to the anti-inflammatory LXA_4_ (201, 202). On the other hand, TNF excess due to high LTA4H activity (TT genotype), resulting in increased LTB_4_:LXA_4_ ratio), leads to activation of the TNF receptor-interacting serine/threonine kinases 1 and 3 (RIP1 and RIP3), mitochondrial reactive oxygen species (ROS), and initiated programmed necrosis (necroptosis) of the macrophages (76). Thus, both the hypoinflammatory (CC) and hyperinflammatory (TT) genotypes of LTA_4_H were associated with poorer outcomes compared to the heterozygous (CT) genotype (201, 202).

The second mechanism for macrophage necrosis is the insufficient clearance of *Mtb*-infected apoptotic phagocytes. *Mtb*-infected macrophages that undergo apoptosis are further phagocytosed by (recruited) uninfected macrophages, a process known as efferocytosis (60). However, if the influx of these uninfected macrophages is insufficient, apoptotic *Mtb*-infected macrophages are not cleared, leading to necrosis (203). While apoptosis of *Mtb*-infected macrophages is known to kill the intracellular bacilli, any surviving bacilli in apoptotic bodies that are not efferocytosed are released during secondary necrosis of the apoptotic bodies (51, 204).

**Figure 4:**
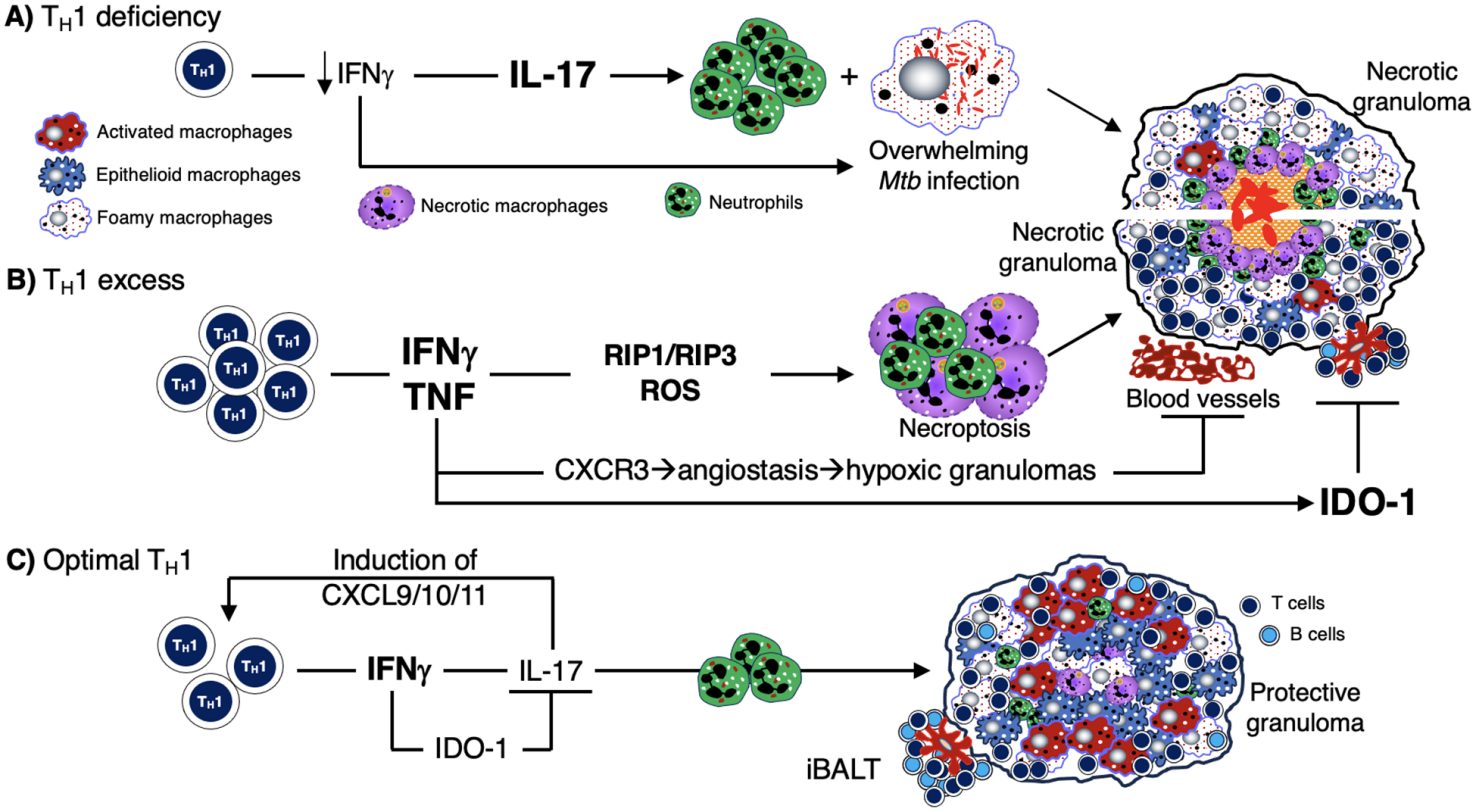
Mechanisms by which deficiency or excess CD4^+^IFNγ^+^ T cells predispose to TB. **(A)** Deficiency of T_H_1 (CD4^+^IFNγ^+^) T cells – as seen in advanced AIDS, genetic defects in the IFNγ-IL-12 axis, and in animal models – has been shown to increase the risk of TB and other mycobacterial infections. This IFNγ deficiency leads to an increase in IL-17 and neutrophilic influx. This combined with a lack of macrophage activation (due to insufficient number of CD4^+^IFNγ^+^ T cells) leads to an overwhelming *Mtb* infection of phagocytes, resulting in an unprotective, necrotic granuloma. **(B)** Excess T_H_1 cell activation and numbers lead to a secondary increase in TNF, which can then induce RIP1 and RIP3 activation, excessive ROS formation, and macrophage necroptosis. The sequence of these events leads to over-inflamed, necrotic, and unprotective granulomas. **(C)** There is ample evidence in both humans and experimental animal models that optimal quantity and temporal influx of *Mtb*-specific CD4^+^IFNγ^+^ T cells are necessary for control ± eradication of *Mtb* infection, in part through activation of macrophages. IFNγ also inhibits IL-17 production from T_H_17 cells which can induce chemokines for CD4^+^IFNγ^+^ T cells. In non-hematopoietic cells, IFNγ also induces IDO-1 production that inhibit excessive IL-17 production (via the catabolized products of tryptophan by the actions of IDO). Hence, IL-17 production is kept in check, limiting the amount of potentially harmful neutrophilic influx. IDO-1, indoleamine 2,3-dioxygenase; IFNγ, interferon-gamma; IL-12, interleukin-12; IL-17, interleukin-17; *Mtb*, *Mycobacterium tuberculosis*; RIP, receptor-interacting serine-threonine kinases; ROS, reactive oxygen species; TB, tuberculosis; TNF, tumor necrosis factor.

The third hypothesized mechanism for the development of caseous necrosis is related to a deficiency or an excess of CD4^+^ T cell response. Deficiency of CD4^+^IFNγ^+^ T cells and insufficient formation of host-protective granulomas (147, 205, 206) results in increased IL-17-mediated neutrophilic influx and overwhelming *Mtb* infection of the macrophages, with both events leading to necrosis of the phagocytes (**Figure 4A**). The mechanism by which an excess of CD4^+^ T cells contribute to necrosis is not clear, but based on experimental evidence, the exuberant IFNγ response induces TNF and other downstream inflammatory mediators (207), causing activation of the aforementioned RIP1 and RIP3, necrotic macrophage death (necroptosis), and release and proliferation of mycobacteria (**Figure 4B**) (76, 208–210). IFNγ also increases CXCR3 (a receptor for several angiostatic chemokines) that may result in granuloma hypoxia and necrosis (**Figure 4B**) (211). Another supporting clue that excess IFNγ (and its downstream effects) may contribute to a detrimental immune response to the host is based on a study showing that IFNγ production by T_H_1 cells is far more important for TB control in the spleen than in the lungs and that increasing the IFNγ-producing capacity by the T_H_1 cells actually exacerbated the lung infection and led to more rapid mortality (77). In contrast to these two extremes of CD4^+^IFNγ^+^ T cell deficiency and excess, we posit that a more precise CD4^+^ T cell response would be more optimal (**Figure 4C**).

A fourth mechanism for the development of caseous necrosis, related to an excess of CD4^+^IFNγ^+^ T cells discussed above, is insufficient negative regulation of IFNγ. *Mtb* induction of IFNγ upregulates the immune checkpoint PD-1 (programmed cell death protein-1), which, in turn, inhibits IFNγ^+^ T_H_1 cells, a form of negative feedback mechanism to prevent excessive IFNγ production (212). The importance of dampening an excessive IFNγ production is evinced by studies showing that PD-1 knockout mice are paradoxically more susceptible to *Mtb* infection (213–216).

A fifth proposed mechanism for the development of necrosis in TB granulomas is the inability of the granuloma to overcome excessive hypoxia (described above), causing cell death and central necrosis in the granuloma. TB granulomas are hypoxic in guinea pigs, rabbits, and NHP (217). Employing dynamic PET imaging using the tracer [^18^F]- fluoromisonidazole, heterogenous degrees of tissue hypoxia were found in the TB pneumonia and around cavities (218). In contrast to the host-protective function of HIF-1α against TB (discussed in Section III.2.2 above), excessive HIF-1α may be harmful. Domingo-Gonzalez et al. (87) showed that when IL-17 was neutralized in *Mtb*-infected mice, there was higher production of HIF-1α, more hypoxic necrotic granulomas, exacerbated inflammation, and increased *Mtb* burden. They further showed that restricting HIF-1α activity in the mice by neutralizing IL-17 reversed both the excessive inflammation and the increased *Mtb* burden (87). While it is not clear how HIF-1α may induce hypoxic necrotic granulomas – since it has known properties that mitigates hypoxia and its detrimental sequelae such as induction angiogenesis and switch to glycolytic metabolism – one possible mechanism is that HIF-1α can induce mucin production and transepithelial resorption of sodium ion and water, resulting in increased mucus in the airways and thus indirectly worsening hypoxia to the lungs and the granulomas (219). Either *Mtb* or hypoxia-induced HIF-1α is known to individually and synergistically induce MMP-1, which can lead to lung tissue destruction and cavity formation *via* its collagenase activity (218). Baay-Guzman et al. (154) found a dual opposing roles for HIF-1α in mice: *(i)* a protective role in early stage TB (≤28 days after infection) through macrophage activation in conjunction with IFNγ and *(ii)* a detrimental role at a later stage of infection through HIF-1α inhibition of apoptosis of foamy macrophages, impairing an effector mechanism by which intracellular *Mtb* are killed.

Finally, type-1 interferons, produced by interstitial macrophages and plasmacytoid dendritic cells, have been found to exacerbate *Mtb* infection, recruit neutrophils, NETs formation, and promote tissue necrosis (220, 221). Not only have type-1 interferons been found to inhibit protective cytokines (IFNγ, TNF, IL-12, IL-1α, IL-1β), but they also contribute to spread of infection and lung inflammation by increasing accumulation of myeloid cells (66, 222). Type-1 interferons have been shown to contribute to necrosis through inhibition of PGE_2_. As noted above, PGE_2_ is known to prevent necrosis of *Mtb*-infected macrophages (61, 64–66). In turn, PGE_2_ (and IL-1) were found to inhibit expression of type-1 interferons (65, 223). It has also been shown that type-1 interferon induction of interleukin-1 receptor antagonist IL-1Ra mediated susceptibility to *Mtb* (224). Despite these host-harmful effects of type 1 interferons during *Mtb* infection, they have also been shown to exhibit host-protective properties in certain conditions; *(i)* co-administration with antimycobacterial chemotherapy; *(ii)* lack of IFNγ signaling from the host; *(iii)* BCG vaccination in certain animal models (65).

### 3.4 Lesion heterogeneity

The physical makeup and host-protective abilities of granulomas can vary greatly. When granulomas form in response to an infection, it gives rise to a trajectory that varies across a spectrum between complete bacterial clearance to uncontrolled infection. Distinct types of TB granulomas exist, all potentially coexisting within a single host infected with *Mtb* (**Table 2**) (138, 152, 225). Several factors can affect granuloma heterogeneity, such as host inflammatory/immune responses as well as differences in physiological-chemical properties of the different regions of the lungs (discussed in Section IV.3 below). Consequently, spatially grouped bacilli may encounter diverse granuloma environments depending on factors such as granuloma type, age, and regional immune response; in turn, *Mtb* can display different phenotypes depending on the microenvironment they are being enveloped (225). This variability leads to a dynamic landscape where some granulomas progress while others regress, even within a single infected individual. This notion of lesion heterogeneity is also supported by reports that sterile (mainly calcified) lesions may coexist with active TB lesions (226).

**Table 2:** Heterogeneity of granulomas and other TB lesions.

Heterogeneity characteristic	Examples
**Morphology**	Lesions can be necrotic, caseous, fibrocaseous, non-necrotizing, neutrophil rich, mineralized, completely fibrotic, or cavitary (7, 138).
**Cellular composition**	Typically macrophages as well as CD4 and CD8 T cells surrounding a necrotic core, but there can also be multinucleated giant cells, neutrophils, dendritic cells, and fibroblasts (4, 138).
**Immune cell response**	Early forming granulomas demonstrate type-2 immune response whereas later forming granulomas demonstrate adaptive T_H_1, T_H_17 and cytotoxic T cell responses (227). Huge variability in numbers and phenotypes of T cells, and range of cytokine profiles and bacterial burdens (138, 234).
**Inflammation**	Proper balance of pro/anti-inflammatory responses is needed to control *Mtb* infection. A more pro-inflammatory response can lead to liquefaction or softening of the caseum (235). A more anti-inflammatory response tends to be associated with an overall better outcome in terms of reactivation as well as long-term prognosis (232). Various inflammatory signatures and killing potentials can be found even within the same granuloma (138, 226, 236). Limitations in the interpretation of these findings include: *(i)* inability to follow the inflammatory phenotype and correlate with culturable *Mtb* in single lesions over time and *(ii)* incapacity to determine whether the inflammation type observed is the cause or effect of the ability or inability of the host to control the infection.

More supporting evidence for this claim comes from a study in which cynomolgus macaques were infected with *Mtb* and their developing granulomas were tracked using computed tomography (CT) combined with deoxy-2-[^18^F]-fluoro-D-glucose (FDG) positron emission tomography (PET) scans before necropsy was performed (226). Based on the PET-CT findings, the metabolic state of individual granulomas showed a dynamic pattern, even within the same animal. Remarkably, their findings revealed that individual granulomas mostly arise from one bacillus of *Mtb* (226). Furthermore, the number of culturable bacteria in the granulomas decreased significantly after time (~15-fold decrease from 4 to 11 weeks). Interestingly, compared to lesions of the NHP at 4 and 11 weeks after infection, animals with latent infection and those with active TB (defined as clinical disease and *Mtb* culture positivity ≥ 3 months after infection) had 10-100–fold less culturable bacteria (226). While the CFU count modestly correlated directly with FDG uptake – a sign of increased glycolysis – it does not necessarily implicate glycolysis as a cause of impaired disposal of *Mtb*; *i.e.*, increased FDG uptake may be a marker for increases of either glycolysis alone or of both glycolysis plus mitochondrial oxidative phosphorylation. Another study using the cynomolgus macaque *Mtb* model found that the lesions showed different immune cell responses based on whether the lesions formed earlier *vs* later (227). Consistent with the prior *Mtb* culture results, earlier forming granulomas in *Mtb*-infected cynomolgus macaques displayed type-2 immune responses, which tend to allow for the survival of *Mtb* within the granuloma, whereas later forming granulomas demonstrate adaptive T_H_1, T_H_17 and cytotoxic T cell responses, which tend to be more effective at killing *Mtb* bacilli and controlling the infection (227). This heterogeneity not only underpins the diversity in infection outcomes between lesions but also complicates treatment efforts. Individual lesions may exhibit varying responses to antibiotics, posing challenges in achieving consistent therapeutic outcomes.

TB is classically divided into “active” vs. “latent” forms, although the current paradigm acknowledges a clinical continuum that also include incipient and subclinical TB (228). Perhaps reflecting these clinical phenotypes is the presence of a spectrum of pathologic lesions that may be seen in human and experimental TB. Following low-dose (~25 bacilli) infection of cynomolgus macaque, about 40% develop active disease whereas 60% develop latent infection (229, 230). In those macaques with active TB, some lesions showed replicating high-burden mycobacteria and necrotic granuloma whereas others showed fewer mycobacteria, more fibrosis, and resolved granulomas, mirrored by PET-CT imaging that demonstrated both metabolically active and metabolically inactive granulomas. Interestingly, even with culture confirmed negativity of NHP with latent infection, both PET-positive and PET-negative lesions may be seen with the caveat that the degree of PET-CT positivity correlates with glucose uptake by the cells and does not necessarily inform the level of control of the *Mtb* infection. In an antibiotic treatment study of pulmonary TB in cynomolgus macaques and in humans, decline in PET activity correlated with reduced bacterial load in both treatment groups and at necropsy in the NHP, with also a reduction in pulmonary pathology (231). Another study showed that a substantial number of humans treated for active drug-susceptible TB remained PET-positive along with positive *Mtb* mRNA in sputa, indicating either subclinical active disease or resolved TB with residual dead *Mtb* (232). These findings show that dividing clinical TB into “active” or “latent” is an oversimplification, and that the dominant type among a spectrum of lesions is likely to dictate the clinical disease (6, 152). Indeed, a recent review categorized TB into eight different states, ranging from elimination by various mechanisms to disseminated disease (233).

### 3.5 Does aerobic glycolysis in TB lesions–granulomas protect against or predispose to TB?

Answering this question is challenging because it is difficult to know in any distinct granuloma that is failing – defined as unopposed *Mtb* replication and worsening pathology – whether the presence of aerobic glycolysis is the cause of the failure or an attempt to rectify the failure. Furthermore, since it has been shown that seemingly distinct granulomas may in fact be different “branches” of the same granuloma (237, 238), the task in answering this question is even more daunting. Nevertheless, we will summarize the reported data.

On the one hand, there are indirect evidence that aerobic glycolysis increases host-susceptibility to TB: *(i)* PET-CT is commonly employed to detect TB lesions in NHP because this avoids the need to sacrifice the animal while obtaining more data in a temporal fashion. While PET-CT has been cited as a radiographic marker of aerobic glycolysis (a Warburg effect) (239), it is not specific for aerobic glycolysis alone since it is only a marker for glucose uptake, which can be seen with increased glycolysis (either aerobic or anaerobic) alone or with increase of both glycolysis and oxidative phosphorylation. Compared to the more TB-susceptible rhesus macaques, the more TB-resistant cynomolgus macaques infected with *Mtb* showed reduced dissemination of infectious granulomas and fewer PET-positive/necrotic lymph nodes (240). While this finding may suggest that aerobic glycolysis increases host susceptibility to TB in the rhesus macaques, one could contrarily argue that the increased PET-CT positivity (increased glycolysis) was an attempt to control the *Mtb* infection in the lymph nodes (240); *(ii)* in cancer cells, there is generally an inverse relationship between glycolysis and autophagy; *i.e.*, increased glycolysis contributes to decreased autophagy (241) and loss of autophagy switches energy metabolism to glycolysis (242); in contrast to cancer cells, the glycolytic byproduct lactate increases autophagy in macrophages (see below); *(iii)* since the lung apices are more alkalotic in upright humans and alkalosis drives aerobic glycolysis (due to increased activity of hexokinase and phosphofructokinase activity (243, 244), key enzymes in the glycolytic pathway), one could also posit that perhaps an increase in glycolysis in the upper lung zones is (partly) responsible for the increased involvement of the upper lung zones in post-primary TB.

**Figure 5:**
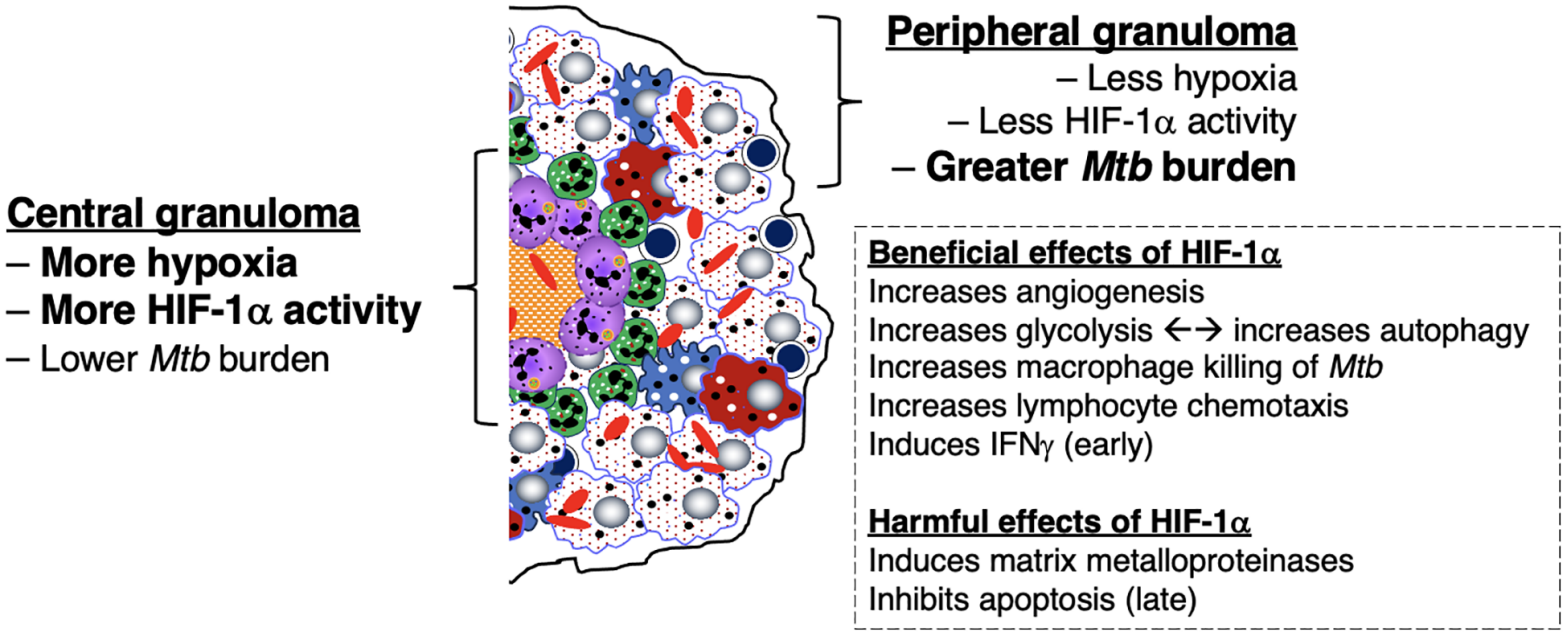
Differential expression of hypoxia, HIF-1α, and downstream effects in the central and peripheral regions of the necrotic granuloma. The central region of a necrotic granuloma is more hypoxic. As a result, there is greater HIF-1α expression with a panoply of downstream effects due to HIF-1 activity. One immune mechanism induced by HIF-1 is aerobic glycolysis, which in turn induces autophagy through the byproduct lactate. Hence, the central necrotic area of an intact human TB granuloma is usually sterile or with low bacterial burden. In contrast, the peripheral region of the granuloma less hypoxic with less HIF-1α induction. In the presence of macrophages that are less effective in killing *Mtb* (as shown by the foamy macrophages), there is less ability to kill *Mtb*, as shown by the greater bacilli number. However, in the presence of activated macrophages and T cells, the peripheral granuloma may also be efficient in either killing or controlling the *Mtb* infection. HIF-1α, hypoxia-inducible factor 1-alpha; IFNγ, interferon-gamma; *Mtb*, *Mycobacterium tuberculosis*; TB=tuberculosis;.

On the other hand, there is a collective strong argument that aerobic glycolysis of macrophages is host-protective against TB: *(i) Mtb* infection of macrophages inhibits aerobic glycolysis and increases fatty acid metabolism by mitochondria; this inhibition of aerobic glycolysis increased mycobacterial growth and decreased the production of certain pro-inflammatory mediators (40, 245–247); *(ii)* phagocytosis is augmented by glycolysis (40, 248); *(iii)* using live *Mtb*, HIF-1α switches macrophage metabolism to aerobic glycolysis which polarizes macrophages to the M1 phenotype (and secondarily to the T_H_1 phenotype) against *Mtb* (162, 163) (**Figure 5**); *(iv)* since the ability of mycobacterial ESX-1 to convert glycolysis to ketosis induces the formation of foamy macrophages – which are known be impaired in controlling intracellular mycobacteria due to decrease of both autophagy and lysosomal acidification (39) – lending credence that glycolysis is host-protective; *(v)* lactate, the byproduct of glycolysis, increases autophagy, possibly through hyperlactylation of PI3 kinase (39, 41, 42); *(vi)* while the aforementioned inverse relationship between protection and aerobic glycolysis in *Mtb*-infected macaques (240) suggests that aerobic glycolysis confers susceptibility, one could argue that the greater protection against TB in the cynomolgus macaques prevented the need for an increase in host-protective aerobic glycolysis; *(vii)* indirect evidence that aerobic glycolysis is host-protective is that calcification of granulomas – a sign of well-controlled TB lesions – is more likely to occur in an alkaline environment, which would also promote aerobic glycolysis; *(viii)* the central parts of necrotic granulomas are considered to be more hypoxic and have greater HIF-1α expression; since HIF-1α increases glycolysis, whose byproduct lactate augments autophagy, this may account for the low *Mtb* number in the central necrotic area of granulomas (**Figure 5**, left-hand side) (161, 163). Because immune cells at the granuloma periphery are less hypoxic and have less HIF-1α activity, there is greater burden of *Mtb* in the outer regions of the granulomas (**Figure 5**, right-hand side). Interestingly, increased aerobic glycolysis may participate in a positive feedback phenomenon as increased autophagy – due to greater macrophage activation – also promotes aerobic glycolysis (43, 44). However, analyzing for the presence of aerobic glycolysis is challenging and requires more testing than simply measuring lactate levels and transcriptional analysis of glycolytic enzymes (40).

### 3.6 Either insufficient or excessive immune response impairs the ability of host granulomas/lesions in controlling *Mtb* infection

There is strong evidence that lack of CD4^+^IFNγ^+^ T cells or IFNγ signaling, as seen in advanced AIDS patients (73, 249) and individuals with genetic mutations of various components IFNγ-IL-12 axis (250–253), respectively are vulnerable to TB and non-tuberculous mycobacterial infections. These clinical evidences are supported experimentally by increased susceptibility of IFNγ-knockout mice to TB (254, 255). Thus, one would predict that genetic disruption of the immune checkpoint PD-1, which increases CD4^+^IFNγ^+^ T cell activation, would enhance the ability of the host to control an *Mtb* infection. Yet, three studies have shown that mice with the *PD-1* gene knocked out, which would increase CD4^+^IFNγ^+^ T cell activation, are more susceptible to *Mtb* infection with not only more severe pathology but greater *Mtb* burden (213, 214, 216). In contrast to the findings with the *PD*-*1* gene knockout mice, wild type mice that were administered blocking antibody to TIM3 (another immune checkpoint) or the *TIM3* knockout mice were more resistant to *Mtb* with reduced the bacilli burden in the lungs compared to their respective control mice (256). Similarly, administration of an anti-CTLA-4 antibody decreased *Mycobacterium bovis* (*M*. *bovis*) Bacillus Calmette Guérin (BCG) burden by one-half log at 6 weeks post-infection (257). Plausible mechanistic explanations for these differences include: *(i)* differential capacity among the various immune checkpoints (PD-1 *vs.* TIM3) to inhibit T effector cell function; *(ii)* the disparity between partial inhibition of immune checkpoints with an antibody *vs.* complete genetic disruption of an immune checkpoint; and *(iii)* separate background strains of mice used (C57BL/6 *vs.* BALB/c) wherein mice that are intrinsically and relatively more immunosuppressed (*e.g.*, the BALB/c background in the *TIM3* gene knockout mice) may be more likely to benefit from immune checkpoint inhibitors.

**Figure 6:**
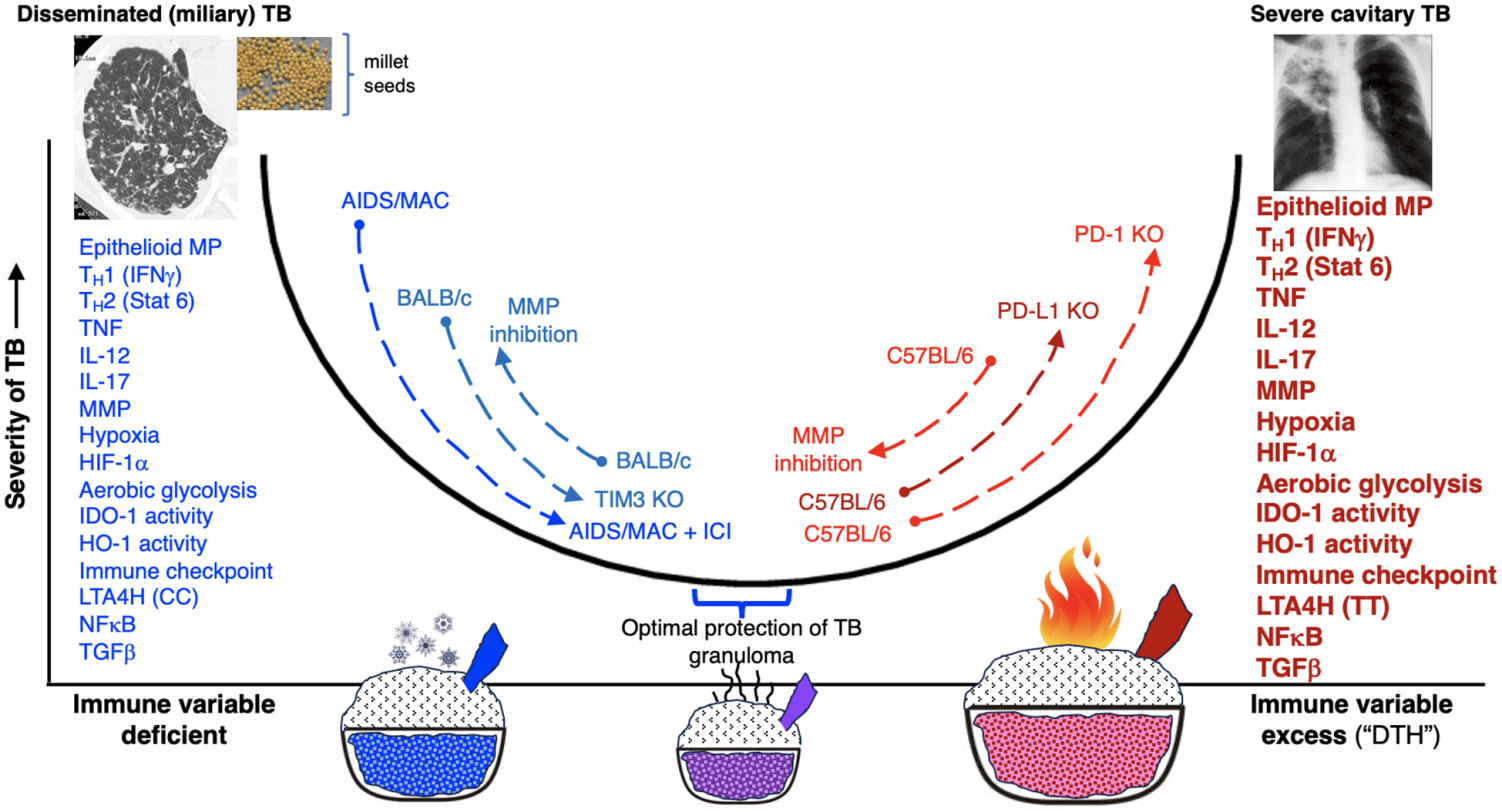
Either excessive or deficiency of molecular or cellular immune elements may predispose to unprotective granuloma or defense against *Mtb*. The diagram illustrates that either deficiency or excessive of specific immune components (x-axis) may result in increased susceptibility and severity of TB (y-axis). Using the immune checkpoint inhibitors as an example, we posit that in conditions where the host is either severely immunocompromised, immune checkpoint inhibition would be beneficial against mycobacterial infection, as exemplified by: *(i)* advanced AIDS patient with severe mycobacterial infection that improved with an anti-PD-1 antibody and *(ii)* TIM3 antagonism or *TIM3* gene knockout mice are protected against mycobacteria in the less immunocompetent BALB/c mice. In contrast, in hosts with better preserved immune function, immune checkpoint inhibition results in a hyperinflammatory state, worsening control of mycobacterial infections, as exemplified by the mice with genetic disruption for *PD-1* or *PD-L1* gene. Perhaps the severity of TB is not as severe in the PD-L1 knockout mice than the PD-1 knockouts because in the PD-L1 knockout mice, PD-L2 is still intact and thus the enhanced immune activation with the genetic disruption of PD-L1 is not as severe as of PD-1. We have also shown other molecular and cellular components in which deficiency or excess predispose to TB. See also text and **Table 2** for further discussion. AIDS, acquired immunodeficiency syndrome; HIF-1α, hypoxia-inducible factor 1-alpha; HO-1, heme oxygenase-1; ICI, immune checkpoint inhibitor; IDO-1, indoleamine 2,3-dioxygenase; IFNγ, interferon-gamma; IL-12, interleukin-12; IL-17, interleukin-17; LTA4H, leukotriene A_4_ hydrolase; MAC, *Mycobacterium avium* complex; MP, macrophages; MMP, matrix *Mtb*, *Mycobacterium tuberculosis*; NFκB, nuclear factor-kappa B; PD-1, programmed cell death protein-1; PD-L1, programmed death-ligand-1; TB, tuberculosis; TGFβ, transforming growth factor-beta; TNF, tumor necrosis factor.

Either deficiency or excess of other molecular and cellular components that impair the host’s ability to control *Mtb* infections are listed in **Table 3**. Thus, we posit that a “modest” activation of these components, at the appropriate times in the course of the *Mtb* infection, are necessary for optimal control of the infection. This “Goldilocks principle” – illustrating the concept of finding the “optimal middle ground” based on the English fairy tale *Goldilocks and the Three Bears* – is also shown graphically (**Figure 6**).

**Table 3:** Deficiency or excess of specific components that impairs host control of *Mtb*.

Component	Deficiency (component beneficial to host)	Excess (component harmful)
**Epithelioid macrophages**	Absolute Stat 6 depletion prevents epithelioid macrophage differentiation, increasing *Mtb* growth in granulomas (36, 37).	Excessive formation may create a “wall” that prevents immune cell influx into granulomas whereas some disruption increases immune access and promote host survival (35).
**T_H_1 (IFNγ)**	Natural examples are individuals with advanced HIV infection (73, 249) or with genetic defect in IFNγ-IL-12 axis (Medelian Susceptibility to Mycobacterial Diseases) who are vulnerable to systemic mycobacterial infections (250, 253) due to CD4^+^IFNγ^+^ deficiency resulting in massive *Mtb* burden and necrotic macrophage death (**Figure 4A**). Deficiency of IFNγ may also augment IL-17 production, inducing neutrophil influx (258). Appropriate IFNγ production induces IDO-1 that inhibits excessive IL-17 production, limiting excessive neutrophil influx (91) (**Figure 4C**).	Excess T_H_1 (IFNγ) results in secondary excess in TNF (207). TNF activates RIP1 and RIP3, induces robust reactive oxygen species, programmed necrosis (necroptosis), and TB cavitation (76, 77) (**Figure 4B**). IFNγ also increases CXCR3 (a receptor for several angiostatic chemokines), causing hypoxia and granuloma necrosis (211). Excess IFNγ-induced IDO-1 (259) can also inhibit iBALT formation.
**T_H_2/Stat6**	T_H_2 cells are required for granuloma formation as T_H_2 deficiency results in failed epithelioid transformation and increased *Mtb* burden (37, 78).	Excess T_H_2 results in insufficient host-protective immunity causing granuloma necrosis, increased delayed-type hypersensitivity to TNF, cavitation, perinecrotic fibrotic capsule, and *Mtb* transmission (79–81).
**TNF**	Mice in which either TNF is neutralized by a monoclonal antibody or have genetic disruption of the 55 kDa TNF receptor are more susceptible to *Mtb* (147, 260). Humans treated with anti-TNF antibody for autoimmune are highly susceptible to TB (141–143).	TNF activates RIP1 and RIP3 and induces production of reactive oxygen intermediates, resulting in activation of BAX, and initiation of a mitochondrial mediated cascade that induces cyclophilin-D-mediated necroptosis (76, 261). TNF also causes anorexia and cachexia (262).
**IL-12**	Deficiency of IL-12 or defect in IL-12 receptor subunit is associated with predisposition to mycobacterial infections (250–253).	Depending on the *Mtb* lineage, the ancestral human IL-12B allele may be associated with more severe TB (suggesting co-evolution of humans and *Mtb*) (136).
**IL-17**	Insufficient IL-17 increases (sequentially) HIF-1α expression, hypoxic necrotic granuloma formation, & severity of TB (87). IL-17 also required for initial formation of protective iBALT (89, 90, 119). IL-17 triggers the production of chemokines that recruit CD4^+^IFNγ^+^ T cells (88).	Excess T_H_17 and IL-17 production increases influx of neutrophils, augmenting granuloma necrosis (91, 92). Sustained & prolonged IL-17 production in iBALT lesions could result in chronic inflammatory & pathologic iBALT (119).
**MMPs**	MMPs facilitate leucocyte recruitment, cytokine and chemokine processing, defensin activation and matrix remodeling (181). Inhibition of MMPs in BALB/c mice was deleterious, resulting in decreased IL-1 and IL-2 expression, premature increase in IL-4, delayed granuloma formation, & more rapid progression of TB (263). In contrast, inhibition of MMPs in C57BL/6 mice was salubrious, resulting in increased fibrosis of the granulomas, decreased leukocyte recruitment (to the granulomas), & decreased numbers of *Mtb* in the lungs & blood in early disease (264).	Excess MMP may lead to immunopathology that leads to *Mtb* dissemination or persistence (181). MMP-9 helps mediate intercellular spread of *M. marinum* (60, 182).
**Hypoxia**	The beneficial, seemingly paradoxical, effects of hypoxia to the host are that hypoxia can: *(i)* impair the growth of *Mtb* as evinced by *Mtb*-infected mice, guinea pigs, & rabbits controlled the TB significantly better when breathing 10% O_2_ (vs. 21% O_2_) (265); *(ii)* induce expression of HIF-1α, augmenting phagocytes to kill *Mtb* in part through impaired ability to activate autophagy (158, 162) (see also HIF-1α below); *(iii)* recruit immune cells to infection site through induction of the chemokine receptor CXCR4 for CD4^+^ T cells (164) & neovascularization (165).	Hypoxia induces necrosis of immune cells in granulomas & of pulmonary tissues (211, 266), causing increased disease severity and provide a niche for *Mtb* (87). Hypoxia also induces secretion of MMP-1 causing lung destruction and cavities (218).
**HIF-1α**	Hypoxia-induced HIF-1α enhances glycolysis to generate ATP quickly, increases angiogenesis (267), coordinates antimicrobial responses of macrophages such as production of inflammatory cytokines (268), and induces greater M1 macrophage and T_H_1 cell phenotypes (154, 162, 163) to kill *Mtb* and other bacteria including pyogenic bacteria and *Mycobacterium avium* (159, 160, 166).	HIF-1α, although induced by hypoxia, has also been shown to induce hypoxic TB granulomas (87), to inhibit apoptosis of *Mtb*-infected foamy macrophages (impairing killing of intracellular *Mtb*) (154), and induce MMP-1 (a collagenase that may cause tissue destruction) (218).
**Aerobic glycolysis**	Aerobic glycolysis skews macrophages and T cells toward the M1 and T_H_1 (IFNγ-producing) phenotypes, augmenting immune cells to control *Mtb* infection (161, 163). Lactate produced from glycolysis increases autophagy and killing of *Mtb* (39, 41)	Indirect evidence that the more TB-resistant cynomolgus macaques have less positive PET-CT lesions (see above discussions). Post-translational modification of proteins by lactate may also lead to excessive inflammation (269).
**IDO-1 activity**	IDO-1 expression in granulomas is found mainly in the rim that surrounds the central necrosis (270). IDO-1 catabolizes tryptophan into kynurenine. Since tryptophan is normally required for *Mtb* growth, absence of IDO-1 would theoretically provide excess fuel for *Mtb* growth (271). Insufficient IDO-1 would increase IL-17, resulting in increased neutrophil influx into and cause necrosis of the granulomas (**Figure 7**).	Increased IDO-1 activity is a predictor of death in *Mtb*-infected hosts (272). Excess IDO-1 activity would decreased proliferation of CD4^+^ and CD8^+^ T cells (132). Reduced remodeling of granuloma resulting in decreased influx of CD4^+^ T cells to the granuloma core (**Figure 7**) (132, 271).. Nevertheless, in the NHP model, (modest) inhibition of IDO-1 results in both reduced *Mtb* burden and disease severity (**Figure 7**) (132, 271).
**HO-1 activity**	HO-1 is protective and promotes granuloma formation in *M. avium* infection (273). In mice, HO-1 deficiency leads to increased susceptibility to *Mtb* infection with increased bacterial loads and mortality due, in part, to failure to mount a protective T_H_1-mediated granulomatous response (274, 275). HO-1 catalyzes the oxidation of heme (as seen with TB hemorrhage) to produce iron, biliverdin, and carbon monoxide that have anti-oxidant, anti-inflammatory, and anti-apoptotic properties to attenuate TB immunopathology (276, 277).	Treatment of macrophages or mice with a HO-1 inhibitor leads to enhanced control of mycobacterial replication (278, 279); it is believed that HO-1 expression increases iron availability in activated macrophages, which benefits *Mtb* growth (276). HO-1 levels correlate directly with the level of active TB but this relationship by itself does not inform whether HO-1 is host-protective or not (280).
**Immune checkpoint**	C57BL/6 mice with genetic disruption of the *PD-1* gene are more susceptible to *Mtb* infection with both severe pathology and greater *Mtb* burden (213, 214, 216). The *PD-L1* gene knockout mice do not have as severe TB than the *PD-1* knockout mice, perhaps because *PD-L2* gene is still intact (**Figure 6**) (213, 281). See above for more detailed discussion.	BALB/c mice with genetic disruption of *TIM3* gene were more resistant to *Mtb* (256). Similarly, administration of an anti-CTLA-4 antibody enhanced mycobacterium-specific T cell proliferation as well as a decrease in *M*. *bovis-*BCG burden (257).
**LTA4H activity**	Individuals with the CC genotype of *LTA4H* possess an anti-inflammatory (decreased LTB_4_:LXA_4_ ratio) and have poorer TB outcome. See above for more detailed discussion.	Individuals with the TT genotype of *LTA4H* possess a pro-inflammatory phenotype (increased LTB_4_:LXA_4_ ratio) and have poorer TB outcome.
**NFκB**	NFκB-mediated responses are critical for restricting bacterial growth in a granuloma (282).	In excess, NFκB may also cause injurious inflammation and cell necrosis (282) and may inhibit apoptosis and autophagy of *Mtb*-infected macrophages (50).
**TGFβ**	In conjunction with IL-23, TGFβ promotes the expansion of T_H_17 cells, which are important for early control of *Mtb* infection (86).	TGFβ negatively impacts IFNγ-producing CD4^+^ T cells in granulomas, impairing control of *Mtb* (83, 101).

CTLA-4, cytotoxic T lymphocyte-associated protein 4; HIF-1α, hypoxia-inducible factor-1 alpha; HO-1, heme oxygenase-1; IDO-1, indoleamine 2,3-dioxygenase; IL-12, interleukin-12; IL-17, interleukin-17; LTA4H, leukotriene A_4_ hydrolase; LTB_4_, leukotriene B_4_; LXA_4_, lipoxin A_4_; MMPs, matrix metalloproteinases; NFκB, nuclear factor-kappa B; PD-1, programmed cell death protein-1; TGFβ, transforming growth factor-beta; TIM3, T cell immunoglobulin and mucin domain-containing protein 3; TNF, tumor necrosis factor.

**Figure 7:**
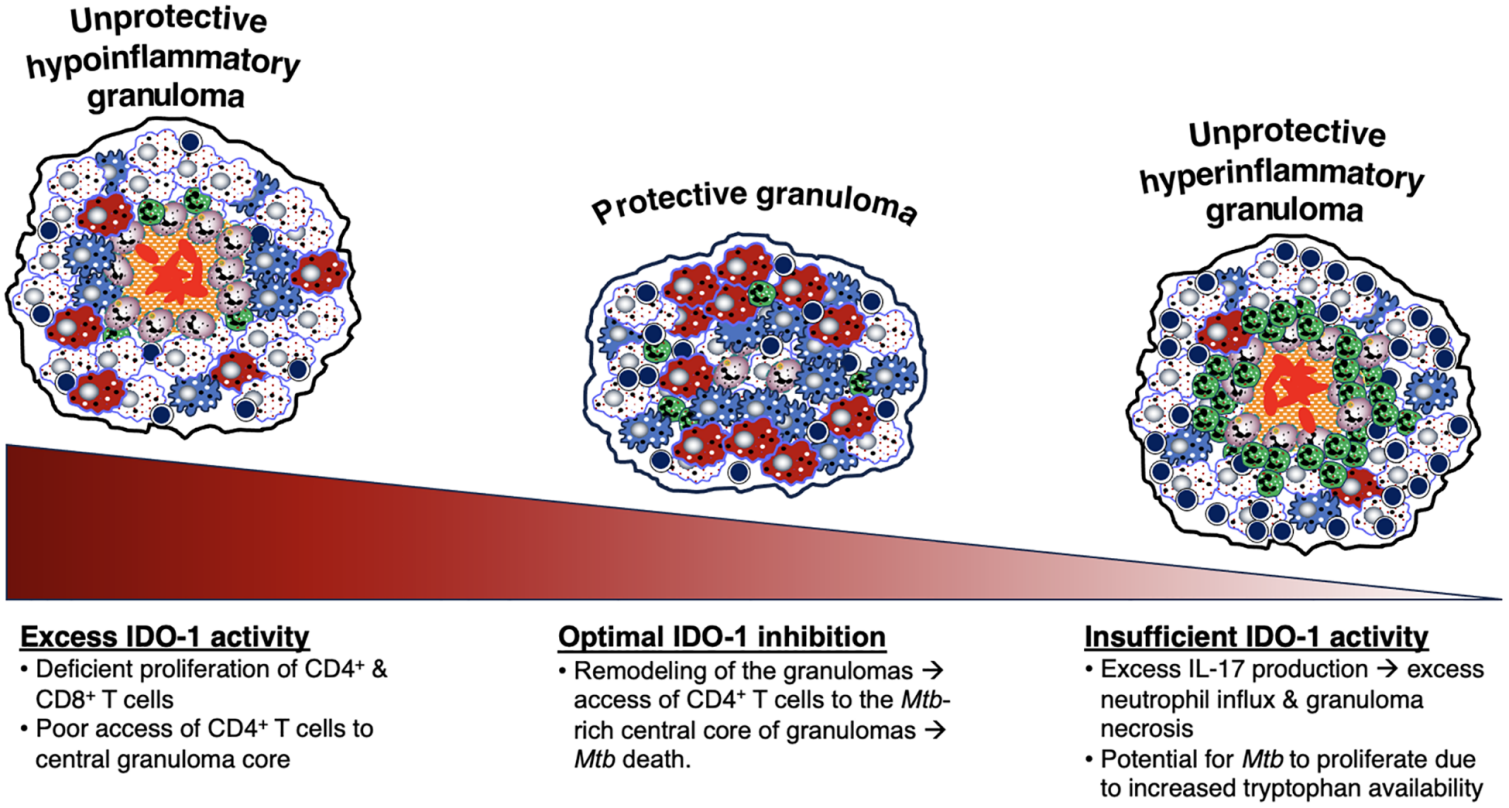
Relative IDO-1 activity helps dictate the level of protection by TB granulomas. IDO-1 activity can also be an indicator of the protectiveness of a granuloma. When there is an optimal level of IDO-1 inhibition, the remodeling of the granulomas allows access of CD4^+^ T cells to the central core of the granuloma, resulting in *Mtb* death. However, excessive or insufficient IDO-1 activity leads to an unprotective granuloma. An excess of IDO activity results in an unprotective, hypoinflammatory granuloma, characterized by deficient proliferation of CD4^+^ and CD8^+^ T cells and poor access of CD4^+^ T cells to the granuloma core, allowing *Mtb* to grow within the center. On the other hand, an insufficient level of IDO-1 activity leads to an unprotective, hyperinflammatory granuloma, characterized by excess IL-17 production and excess neutrophil influx and granuloma necrosis. IDO-1 is also responsible for the catabolization of tryptophan (a molecule necessary for *Mtb* growth) into kynurenine. A deficiency of IDO-1 would in turn increase levels of tryptophan and allow for more *Mtb* growth. IDO-1, indoleamine 2,3-dioxygenase; IL-17, interleukin-17; *Mtb*, *Mycobacterium tuberculosis*;.

## 4 Characteristics of human TB and granulomas

In humans, active TB is classically divided into primary TB and post-primary TB, the latter known as reactivation TB, usually located in the lungs after establishment of systemic host-protective immunity in primary TB (283). Since *Mtb* is acquired through inhalation of *Mtb*-containing droplet nuclei less than 5 µm that reach the terminal bronchioles and alveoli, the granulomas in primary TB are located more commonly in the lower lung zones where the ventilation is greater (284). In contrast, the location of post-primary TB is typically more prevalent in the upper lung zones, especially the lung apices but also the superior segments of the lower lobes. These differences suggest that the immunological correlates of protection of primary TB and post-primary TB may be quite different as perhaps evinced by BCG protection against primary TB but less successfully with post-primary TB (285).

### 4.1 Self-limiting primary TB and primary progressive TB

In immunocompetent individuals, primary TB – most commonly affecting the lungs but may present as extrapulmonary TB – typically remains asymptomatic and resolves spontaneously. It is most often detected radiographically as a Ghon lesion (a sequela of untreated primary TB characterized by a lung nodule comprised of caseous granulomas surrounded by a fibroblastic rim), a Ghon complex (the combination of a nodule with associated draining mediastinal lymphadenopathy), or a Ranke complex (characterized by calcification of the Ghon complex) (286). On imaging, such lesions may be suspicious for lung cancer prompting surgical removal, but diagnosed histopathologically as TB-related (287). A relatively small number of bacilli adapt to the granuloma micro-environment and persist as asymptomatic latent infection. Autopsy studies revealed that granulomas in latent TB tissues exhibited lower cellularity and inflammation but displayed more prominent sclerosis, fibrous encapsulation, and calcification compared to their active counterparts (152). Histological analysis of latent TB lesions from individuals who died of non-TB-related causes has revealed caseous and fibrocaseous characteristics (288).

Ghesani and co-workers (289) reported the PET-CT findings of five household contacts of active TB patients before and during treatment for latent TB infection. These five individuals had no evidence of active TB and asymptomatic (“latent”) TB infection was established based on positive QuantiFERON testing and negative chest radiographs. This report showed that: *(i)* FDG uptake was increased in the thoracic lymph nodes in four of the five contacts; *(ii)* on repeat PET-CT testing while on treatment for latent TB infection, the FDG uptake decreased or became negative in three of the four and no change in one with initial mild FDG uptake; *(iii)* in the one patient with initial negative FDG uptake in the chest, there was evidence of remote latent TB infection as evinced by calcified granuloma in the lung parenchyma and ipsilateral calcified hilar lymph node (Ranke complex); *(iv)* the QuantiFERON results correlated highly with the intensity of the FDG uptake, indicating the PET positivity likely reflected a type 1 (CD4^+^IFNγ^+^) immune response (289).

Some individuals, perhaps with sub-optimal and not necessarily severely immunocompromised immune function may develop primary progressive TB. Except for a history of a relatively recent active TB exposure and the location of disease (more likely to be mid to lower lung zones), such patients are essentially indistinguishable from those with post-primary TB.

### 4.2 Post-primary TB

**Figure 8:**
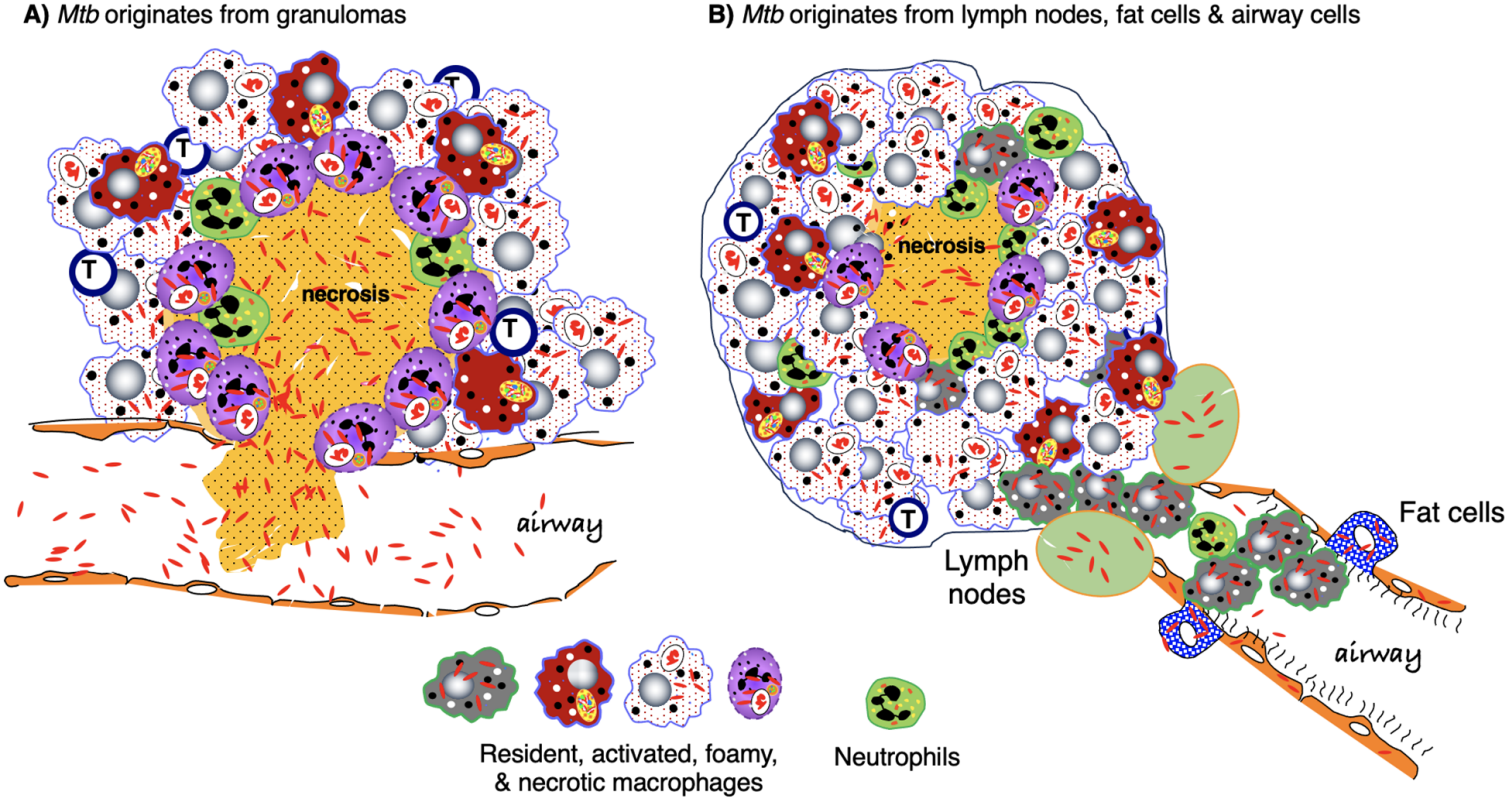
Origin of *Mtb* and early events in the development of post-primary TB. **(A)** An often-cited description for the evolution of post-primary (reactivation) TB is that immune dysfunction results in progressive necrosis and liquefaction of the granuloma, exuberant multiplication of dormant *Mtb*, disruption of the integrity of the granuloma wall, and spillage *Mtb*-filled necrotic granulomatous content into the airways. Pneumonia forms due to endobronchial spread of granuloma content. **(B)** An alternative description of post-primary TB is based on the paradigm chronic stable granulomas are mostly sterile and that the dormant *Mtb* that “reawaken” originated from lymph nodes. fat cells, and airway epithelial cells causing a bronchogenic lipoid pneumonia. Granulomas may form around pockets of necrotizing pneumonia. As discussed in the text, it is plausible that both reactivation models occur. *Mtb*, *Mycobacterium tuberculosis*; TB, tuberculosis.

Post-primary TB accounts for the majority (≥80%) of active TB cases. In the 19th and early 20th centuries, pathologic analyses of human TB tissues were commonly performed on subjects with varying profiles of disease severity. These studies were conducted prior to the discovery of effective antibiotic therapy and thus also reflect the natural history of TB (134, 288, 290–292). Dr. Robert Hunter and his colleagues have critically reviewed these older literature as well as microscopically examined treatment-naïve TB lung specimens (287, 293–297). Two characteristic lesions of human pulmonary TB emerged: caseating granulomas and tuberculous pneumonia (**Figures 8A, B**) (293). Caseating granulomas were reflective of primary TB, which may be self-limiting or progressive (**Figure 8A**). While post-primary TB may begin with degeneration of primary granulomas (**Figure 8A**), some contend that it starts as a nascent pneumonic process which then spreads endobronchially resulting in airway obstruction and lipoid pneumonia, the latter due to the presence of lipid-laden (“foamy”) macrophages and DEC-205^+^ dendritic cells infiltrating the airway lumina and alveolar spaces (**Figure 8B**) (33). However, because most surgically resected or autopsy TB cases represent advanced disease, it is challenging to determine with certainty whether endobronchial spread is a primary or secondary process, or a combination of both. The early inflammation that occurs with this post-primary pneumonic process was largely attributed to a Type IV hypersensitivity reaction to *Mtb* antigen and not to an increase in the number of viable *Mtb* since the early stages of this pneumonic process were very often paucibacillary (133, 298, 299). The formation of necrotic granulomas due to host-mediated bacillary destruction rather than proliferation is supported by recent RNAscope and immunohistochemistry studies (300). In this study, a significant amount of *Mtb* mRNA and Ag85B was detected in human necrotic granulomas in the lungs and lymph nodes of a confirmed TB patient, despite being Ziehl-Nielsen-negative (300).

These findings are further corroborated by another study that characterized the micro-anatomical environment of human TB lung tissues using micro-computed tomography (µCT), histopathology, and immunohistochemistry to determine the three-dimensional shape of the TB granulomas and their spatial relationship to airway and vascular structures (237, 238, 301). Consequently, unlike the typical two-dimensional description of TB granulomas as round discrete structures, they found that human necrotic TB granulomas demonstrated cylindrical, branched morphologies which are connected to the airways and shaped by the bronchi, supporting the bronchogenic spread of *Mtb*. Another clinically relevant finding is that the lack of vascularization within obstructed bronchi likely resulted in further necrosis poor antimicrobial penetration of affected lung segments, and the development of functional drug resistance (237). Additionally, utilizing histopathology and genome-wide transcriptomic analysis, immunological profiling of human lungs surgically resected for recalcitrant active TB showed considerable variation in T cell influx in different granulomas as well as significant differences in gene expression of the inflammatory response, immune cell trafficking, and cell mediated immune response (5).

The post-primary pneumonic process can either resolve spontaneously with resolution of the airway obstruction (thought to occur in the majority of cases) or develop necrosis and a caseating pneumonia (in ~5% of cases) (**Figure 8B**). One *Mtb* component that may be responsible for the caseating pneumonia is the glycolipid TDM (185, 188). If caseous necrosis develops – often linked to a rapid surge in *Mtb* numbers (133, 297) – the infection may progress in one of two ways: either through the formation of a post-primary granuloma around the caseous pneumonia, attempting to contain the infection, or through cavitation, which leads to even greater *Mtb* replication and facilitates their expulsion into the airways, enabling person-to-person transmission. The pathogenesis of cavitary disease may arise not only from the caseating necrosis associated with the pneumonic process but also from the degradation of fibrous tissue at the periphery of secondary granuloma by MMP-1 and MMP-9 (302–306). Additional factors for cavitation include pulmonary infarction resulting from vasculitis (287, 294), and an excessive immune response (285, 307, 308). Interestingly, the lack of necrotizing granulomas in most standard mouse strains may be linked to the absence of lung expression of the mouse ortholog of MMP-1 (303, 304).

### 4.3 Where do the *Mtb* originate from in post-primary TB?

**Figure 9:**
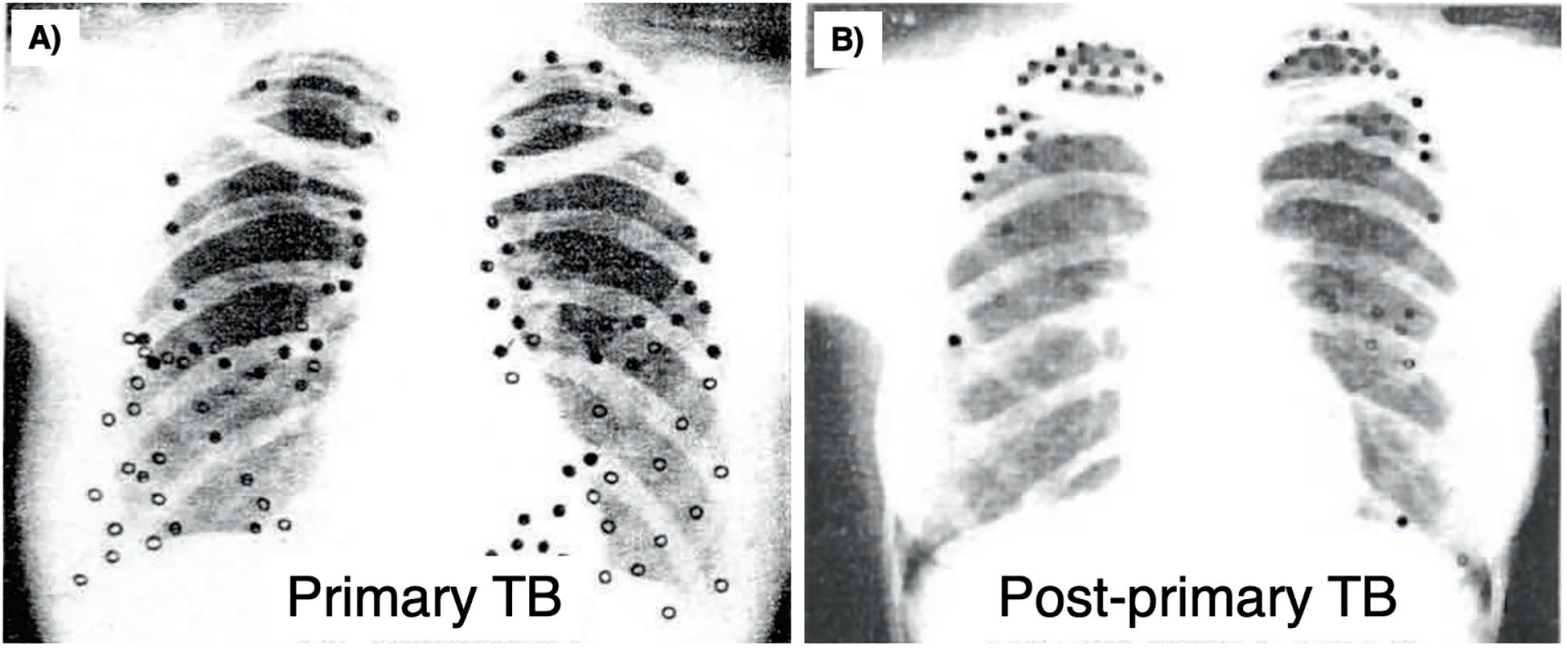
Distribution of lesions in primary and post primary TB. The localization of lesions of **(A)** primary and **(B)** post-primary TB of several people were plotted on an X-ray. Each dot marks the location of an individual’s lesion. The distribution of primary TB is consistent with chance distribution of air-borne infection while most post-primary lesions were in the upper lobes, especially the apical segments. Reprinted with permission of the American Thoracic Society. Copyright ^©^ 2024 American Thoracic Society. All rights reserved. Medlar E.M. The pathogenesis of minimal pulmonary tuberculosis: A study of 1,225 necropsies in cases of sudden and unexpected death. Am Rev Tuberc 1948; 58: 583-611. The American Journal of Respiratory and Critical Care Medicine is an official journal of the American Thoracic Society. TB, tuberculosis.

The precise location of dormant *Mtb* before their reactivation, leading to post-primary TB, remains a topic of debate. Some argue that dormant *Mtb* residing within primary granulomas break out as the integrity of the granulomas declines due to waning immunity (**Figure 9A**). Subsequently, *Mtb* enters the airway lumina, leading to endobronchial spread. Alternative evidence suggests that *Mtb* originates from normal-appearing lung tissues, fat cells, and thoracic lymph nodes since primary granulomas are noted to be sterile after five years (21, 294, 309). However, unless all innumerable microscopic granulomas in the lungs of primary TB can be individually dissected and cultured – logistically an untenable task – it is impossible to exclude primary TB granulomas as potential sources of reactivated *Mtb* during development of post-primary TB. Furthermore, both hypotheses are not necessarily mutually exclusive, leading to a reasonable supposition that both quiescent lung granulomas and other tissues (fat cells, lymph nodes) are potential sources of reactivated *Mtb*. Following cavitation, endobronchial spread may occur to all other parts of the lungs (310).

**Figure 10:**
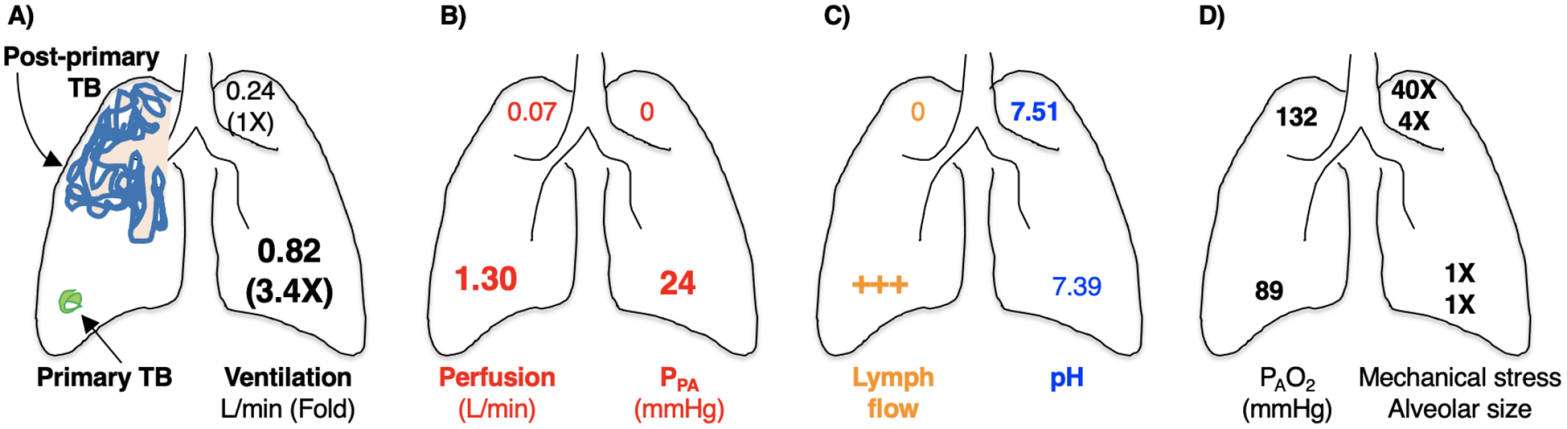
Anatomical-physiological-chemical mechanisms by which post-primary TB is more common in the upper lobes of the lungs. Based on differences in the physiological-chemical properties between the upper lobes and lower lobes, primary *Mtb* lesions located in the upper lobes, while fewer than that in the lower lobes, are more likely to reactivate. **(A)** Diagram showing that primary TB is located mostly in the lower lung zone (green circular structure that represents a calcified granuloma – Ghon complex). This distribution of primary TB is due to greater (3.4X) ventilation in the lower lobes than the upper lobes of an upright human. **(B)** In the upright human, there is decreased lung perfusion pressure due to lower pulmonary artery pressure in the upper lobes. This decreased perfusion will result in decreased influx of immune cells and soluble mediators to the upper lobes in the upright position. **(C)** Lymph formation and flow are also significantly decreased in the upper lobes, resulting in increased accumulation of mycobacteria and antigens in the upper lobes and contributing to increased hypersensitivity reactions and lung damage. pH in the upper lobes is more alkalotic which induces glycolysis and, in turn, increases autophagic clearance of *Mtb* by the macrophages. **(D)** The P_A_O_2_ is also significantly greater in the upper lobes than the lower lobes, which favors *Mtb* growth. The mechanical stress and alveolar size of the upper lobes are also greater (by 40X and 4X, respectively) than the lower lobes, predisposing this area to the development of cavities, especially with large inspiratory efforts. *Mtb*, *Mycobacterium tuberculosis*; P_A_O_2_, partial pressure of alveolar oxygen; TB, tuberculosis.

Most primary TB lesions reside in the lower lung zone (**Figures 9A**, **10A**) because inhaled *Mtb* are preferentially deposited in the more ventilated lower lung zones. However, primary TB lesions can also form in the upper lung zones, albeit less frequently (**Figure 9A**) (294, 298). Nonetheless, post-primary TB is more common in the upper lobes (especially apices) and superior segments of the lower lobes (**Figures 9B**, **10A**). The exact reason for the differing lung distribution between primary TB and post-primary TB remains unclear, as it seems unlikely to be solely attributed to the initial residence of dormant *Mtb*. We suggest that this locational disparity arises from the preferential reactivation of primary lesions in the upper lung zones, influenced by fundamental physiological and chemical principles.

First, while there is less ventilation to the upper lung zones in the upright position in humans, there is much less perfusion in the upper lung zones (**Figure 10B**). The relative overall decrease in blood perfusion to the upper lung zones will also result in decreased influx of both immune cells and soluble factors such as antibodies and complement components (311, 312). Second, because the lung apices have decreased capillary perfusion pressure in the upright human, there is decreased lymphatic fluid formation in the upper lung zones, resulting in reduced lymphatic drainage of *Mtb* antigen/debris removal to lymph nodes and reduced T cell priming (**Figure 10C**) (313). Third, there is relative alkalosis in the upper lung zones compared to the lung bases (~pH 7.51 vs. 7.39, respectively) due to increased ventilation:perfusion ratio in the upper lung zones (314). Alkalosis promotes aerobic glycolysis over oxidative phosphorylation (315, 316) and this increase in glycolysis can hinder autophagic flux (241), impairing a key mechanism for killing intracellular *Mtb*. Supporting this hypothesis, experimental TB in rabbits predominantly affects the dorsal regions of the lungs; however, when the rabbits are kept in a vertical position, the infection primarily targets the superior parts of the lungs (317, 318). Fourth, the relatively greater O_2_ delivery to the upper lung zones (**Figure 10D**) creates a more hospitable environment for *Mtb* replication (313, 319). The reason that there is greater O_2_ tension in the upper lobes is that perfusion by deoxygenated blood arising from the pulmonary artery in a low pressure right ventricular circulation is surpassed by oxygenated blood flow from the bronchial artery, which is a high-pressure left ventricular circulation; *i.e.*, compared to the lower lobes, there is relatively less O_2_ extracted from the upper lobe alveoli by the red blood cells in the upright human. This notion is supported by evidence that in marginal human donor lungs, there is significantly better oxygenation to the upper lobes than the remaining lungs, as measured by the ratio PaO_2_:FiO_2_ (partial pressure of O_2_ in the blood:inspired O_2_ concentration) (320). One piece of supporting evidence for this mechanism is that *Mtb*-infected guinea pigs breathing 10% oxygen exhibit less severe TB pathology compared to control animals breathing ambient air (265, 317). Conversely, *Mtb*-infected mice breathing 60% O_2_ had significantly higher burden of viable *Mtb* in the lungs than control mice breathing 20% O_2_ (317).

### 4.4 What causes pulmonary cavitation?

There are two prevailing models regarding the formation of TB cavities in the lungs, each corresponding to the two main models of post-primary TB development. It is likely that both models occur with varying frequencies within individuals. The first model suggests that a stable granuloma harboring dormant *Mtb* becomes destabilized due to “immunosuppression,” leading to liquefaction of the central caseous necrosis and subsequent multiplication of *Mtb*. The contents of the cavity then erode into an airway, allowing air to enter the emptying granuloma and expanding the space. The second model proposes that post-primary TB initially presents as bronchogenic lipoid pneumonia, which then undergoes necrosis. This triggers a secondary granulomatous reaction, which forms around the necrotizing pneumonia, resulting in cavitation due to the necrosis and expectoration of the necrotic material. One finding that challenges both models is that air analysis reveals most cavities contain lower oxygen levels and higher carbon dioxide levels than those measured in the airway lumen (321). However, it is plausible that intermittent or progressive obstruction of the airway emanating from the cavity could lead to hypoxia and hypercarbia within the cavity lumina. Once cavities form, further necrosis and disruption of the cavitary walls may allow extracellular mycobacteria to access the blood and lymphatic vessels, as well as the airways, facilitating their spread to other individuals through aerosolization (322). Tuberculous cavities typically harbor high concentrations of bacilli, and their presence heightens infectiousness due to their communication to the airways and subsequent aerosol transmission of *Mtb*. While necrosis is a prerequisite for cavitation, it alone is insufficient to ensure its development.

Recently, a study compared the cell-specific gene expression profiles of sarcoidosis granuloma (usually not associated with necrosis) and human TB granuloma (often associated with necrosis). Compared to sarcoidosis, the most upregulated gene in TB was the collagenase matrix metalloproteinase-1 (MMP-1) (305). MMP-1 is a collagenase that is essential for cavity formation through the degradation of the fibrous extracellular matrix that surrounds granulomas (304). Infection with *Mtb* induces MMP-1 and MMP-9 production in macrophages (302). During the early stages of granuloma formation, MMP-1 expression increases in the surrounding epithelial cells and leukocytes (181). While MMPs facilitate leucocyte recruitment, cytokine/chemokine processing, and defensin activation, excess MMP activity may lead to damaging immunopathology including granuloma destruction and pathogen dissemination or persistence (181, 303). Inhibition of MMP with a broad spectrum MMP inhibitor yielded conflicting results, but this may be due to timing of the inhibition in relation to the *Mtb* infection (263, 264). Early MMP inhibition (at the time of *Mtb* infection) with a broad spectrum MMP inhibitor (BB-94) in mice resulted in decrease in pro-inflammatory cytokines (IL-1 and IL-2), premature increase in IL-4, delayed and smaller granuloma formation, and more rapid progression of disease characterized by small lung nodules suggestive of miliary TB (263). In contrast, delayed administration of BB-94 (18 days after *Mtb* inoculation) resulted in increased collagen deposition within early granulomas with decreased *Mtb* burden (264).

Mechanical factors may also account for why TB cavities are more common the upper lung zones. In the upright human, there is significant increase in both physical stress and alveolar sizes in the upper lung zones during inspiration, ~40 times and ~4 times greater, respectively, than the lower lung zones (**Figure 10D**). These physical differences may subject the upper lung zones to increased physical stress and along with actions of various metalloproteinases produced by immune cells that degrade lung tissues, increase vulnerability to cavitary formation. Based on these plausible pathogenic mechanisms, it has been suggested that historical TB treatment of enforced bed rest may be an effective adjunctive remedy for TB (312).

Animal models are essential for studying TB pathogenesis and developing treatments and vaccines. As with all disease models, it is vital to select species that replicate key characteristics of human TB, including the development of caseous necrosis surrounded by a collagen rim, hypoxia, both intracellular and extracellular populations of bacilli, liquefaction, and cavity formation (152). In this context, we will first discuss select animals with natural *Mtb* complex infections, such as cattle and elephants, followed by a review of experimental animal models including zebrafish, mice, guinea pigs, rabbits, and non-human primates (152, 266, 323–327).

## 5 Characteristics of natural TB infections of animals

### 5.1 Cattle

Cattle are a natural host of *Mycobacterium bovis*, an *Mtb* complex organism (328). While cattle can be experimentally infected with *Mtb*, TB is less severe (329). *M. bovis* also infects many other domesticated and wild animal species (328, 330). Cattle have several advantages in TB vaccine research – the disease progresses slowly, the granulomatous reaction are similar to that seen in humans, and vaccination in neonatal calves confers protection but wanes over time (331–334). Disadvantages include their large size/high cost in experimental housing (326) and post-primary TB is not a characteristic of them (294).

Granulomas in tuberculin reacting cattle (due to *M. bovis*) may be uncommon to rare (1-2%) when tissues are examined in a slaughterhouse but are commonly found (>70%) when analyzed using 5 mm lung slices (335). Wangoo et al. (336) have classified bovine TB granulomas in experimentally infected calves with *M. bovis* into four separate stages of severity based on size and microscopic morphological characteristics such as cellular composition, necrosis and fibrosis. Similar to humans and NHP, all stages of granulomas in this *M. bovis* infection model may be seen simultaneously in the same animal (336, 337).

As in human TB, CD4^+^ and CD8^+^ T cells are important in host immunity to bovine TB. In most stage I/II lesions of bovine TB, CD8^+^ and CD25^+^ T cells were found in the rim of the granulomas whereas CD4^+^ T cells were distributed in both the lymphocytic rim and center of the granulomas (337). γδ T cells were interspersed among the macrophages, leading to the speculation that CD4^+^ and γδ T cells – observed in granulomas within the first 21 days after experimental *M. bovis* infection in cattle (338, 339) – both play a role in the maintenance and the maturation of the granulomas as well as a host-protective role in respiratory mucosal immunity (340, 341). In stage III/IV lesions, all the examined T cell populations were present among the mononuclear component of the granulomas. The clinical and pathologic features of *M. bovis*-infected cattle that mimic human pulmonary TB are described in **Table 4**.

**Table 4:** Comparison of the features of natural and experimental animal TB with human pulmonary TB.

Present in animals?	Human TB Features							
	Clinical features			Pathologic features				
	Latent infection	Primary progressive TB	Reactivation TB	TB pneumonia		Non-caseating granuloma	Caseating granuloma	Cavitary disease
**Cattle**	Yes (461–465)	Yes (334, 336–340, 461)	Not reported “naturally” but experimentally with recovery of *M. bovis* with or without treatment of animals with the “mycobacterial promoter resuscitation factor B (RpfB) protein” (462–464).	Yes (lipoid pneumonia) (29)		Not reported	Yes (336)	No (29, 266, 323)
**Elephants**	Yes (352, 357, 359, 466)	Possible but unable to distinguish readily with reactivation TB.	Yes (352, 467)	Yes (349, 358)		Not reported	Yes (345, 347, 348, 354, 358)	Yes (348, 358)
**Zebrafish**	Yes, with low-dose *M. marinum* infection (468)	Yes, with high-dose *M. marinum* (369) or with “cluster I” strain (isolated from humans with fish tank granuloma). Chronic disease with longer survival of the zebrafish is seen with infection with “cluster II” strain of *M. marinum* (469).	Yes, of latent infection with gamma-irradiation (468)	N/A		Yes (361)	Yes. With time, the central necrosis of the granulomas may be surrounded by a cellular and/or fibrotic cuff (168, 369).	N/A
**Mice**	Used antibiotics to suppress *Mtb* but not kill them with ability to reactivate (Cornell model) (384).	Yes (34, 185, 374, 376, 383, 386).	Yes, with use of non-sterilizing antibiotic regimen (384) and with (premature) termination of antibiotic regimen (376).	Yes (295, 387).		Yes (383).	Yes, with the C3HeB/FeJ (Kramnik) mouse strain (34, 376), the CBA/J strain (470), or intraperitoneal immunization with *Mtb* and rechallenge (185).	Yes, in certain strains (C3HeB/FeJ and CBA/J) (386, 387).
**Guinea pigs**	Yes, with use of antibiotics to suppress *Mtb* (402, 403).	Yes (325, 395, 401, 407, 471, 472).	Yes (405).	Yes (406, 473).		Yes (472).	Yes (325, 472).	Yes, sporadically (325, 372, 471).
**Rabbits**	Yes, in the TB-resistant New Zealand white rabbits infected with *Mtb* CDC1551 (425).	Yes (420, 474).	Yes, glucocorticoid was used to cause reactivation TB in New Zealand white rabbits with latent TB infection (425).	Yes (474).		Yes (425).	Yes (323, 475).	Yes (323, 420, 431).
**Mini pigs**	Yes (435, 436).	Yes (435, 436).	Unknown but potentially yes.	None reported		Yes (435, 436).	Yes (435, 436).	None reported.
**Goats**	Yes. Goats inoculated with *M. bovis* in the airways did not develop clinical signs of infection when euthanized 5 months later; there were also *M. bovis* cultured from thoracic lymph nodes, calcified lesions in the lungs, and skin test confirmed bovine TB (438).	Yes (437, 476).	Yes (477).	Yes, with foamy macrophages (478).		None found.	Yes (437, 438, 477, 479).	Yes (437, 477).
**NHP**	Yes, in rhesus and cynomolgus macaques (229, 452, 480).	Yes, in rhesus and cynomolgus macaques (226, 240, 451, 481–485). Yes, in marmosets (447, 486).	Yes, in both rhesus and cynomolgus macaques (229, 452, 480). Reactivation from latent infection can occur but is rare (<5%) (487).	Yes, in rhesus and cynomolgus macaques (226, 483, 487). Yes in marmosets (447).		Yes, in rhesus and cynomolgus macaques (482, 487). Yes, in marmosets (447).	Yes, in rhesus and cynomolgus macaques (451, 452, 484). Yes, in marmosets (447).	Yes, in rhesus and cynomolgus macaques (229, 451, 452, 485). Yes, in marmosets (447, 486).

Differences between Rhesus and Philippine cynomolgus macaques with TB								
Rhesus macaques	Cynomolgus macaques
More susceptible to *Mtb* with higher bacterial burden, greater signs of clinical disease, and slower progression of TB (240).	More resistant to *Mtb* with fewer symptoms, reduced dissemination of granulomas and slower progression of disease (240).
More gross pathology (240).	PET-CT shows reduced dissemination of granulomas, slower increase in lung inflammation, and fewer PET-positive/necrotic lymph nodes than rhesus macaques (240). Immunohistochemistry showed greater influx of macrophages, B cells, and T cells in most of the granuloma types than rhesus macaques (488).
More reliable model for active disease (240).	More reliable model for latent infection (452).

### 5.2 Elephants

Elephants are divided into African elephants (*Loxodonta africana*) and Asian (Indian) elephants (*Elephas maximus*). While TB in elephants is mostly due to *Mtb*, *M. bovis* (342–345) and *M. caprae* (346) have been reported. TB in elephants has been identified more commonly in Asian elephants than African elephants, possibly due to greater contact between the former and humans. However, it is not known whether Asian elephants are intrinsically more susceptible (347, 348).

TB has been observed in both wild (349–351) and captive elephants (344, 352). Spread among elephants and inter-species infection between humans and elephants (likely bidirectional) have been either strongly suspected or well-documented (353–357).

Heterogenous TB granulomas are also found in elephant TB. The main pathological lesions of elephant TB occur in the lungs and thoracic lymph nodes (358). Gross anatomical features include non-caseating and caseating granulomatous nodules as well as calcified caseous and cavitary lesions. Histopathologic features include epithelioid granulomas with giant cells in both lymph nodes and lung lesions as well as caseous and necrotizing granulomatous pneumonia in more severe cases (358). One study showed that of nine Asian elephants that died from TB, six had poorly-formed granulomas in the lungs with low to moderate number of lymphocytes, comprised mostly of B cells (359). However, the other two elephants had discrete and well-demarcated granulomas and greater number of T cells. The clinical and pathologic features of *Mtb*-infected elephants that mimic human pulmonary TB are described in **Table 4**.

## 6 TB Granulomas associated with experimental infections

### 6.1 Zebrafish

The primary limitations of zebrafish in experimental TB are that they do not have lungs and cannot be infected in a sustained fashion with *Mtb* due to lower temperature requirements of zebrafish. Instead, *M. marinum* is used as a surrogate because it naturally inhabits zebrafish, is genetically similar to *Mtb*, and causes TB-like diseases in poikilothermic animals (76, 261, 360, 361). Nevertheless, both the embryonic-larval and adult zebrafish models have several advantages in understanding mycobacterial pathogenesis: *(i)* large-scale screening studies can be attempted since they are small and easy to reproduce and raise; *(ii)* their transparency in the embryonic and larval stages allows their internal structures to be visualized temporally without the need to sacrifice them; *(iii)* zebrafish embryos have a temporal separation between the earlier development of innate immunity (macrophages and neutrophils) and later development of adaptive immunity (lymphocytes), allowing the study of only innate immunity during the initial stages of mycobacterial infections; *(iv)* hypoxic or non-hypoxic granulomas can be induced according to the infection site – *via* inoculation of *M. marinum* in poorly or richly vascularized areas, respectively – in the larva model; *(v)* in the advanced model, acute or chronic/latent infection may be induced based on the infectious dose (4, 362–370). The clinical and pathologic features of *M. marinum*-infected zebrafish that mimic human pulmonary TB are described in **Table 4**.

### 6.2 Mice

Mice are a tractable animal model for TB because they are relatively easy to handle, inexpensive, require little space, have similar immune components to humans, and there is an expansive array of immunological reagents available. Furthermore, there is the ability to genetically engineer mouse strains to elucidate the role of specific genes with *Mtb* infection; *i.e.*, “knock-out” and “knock-in” mouse strains through targeted disruption of specific genes (266, 323, 326, 371, 372). These advantages allow for assessment of large-scale drug screening and vaccine efficacy testing, in addition to monitoring disease endpoints following *Mtb* infection.

**Figure 11:**
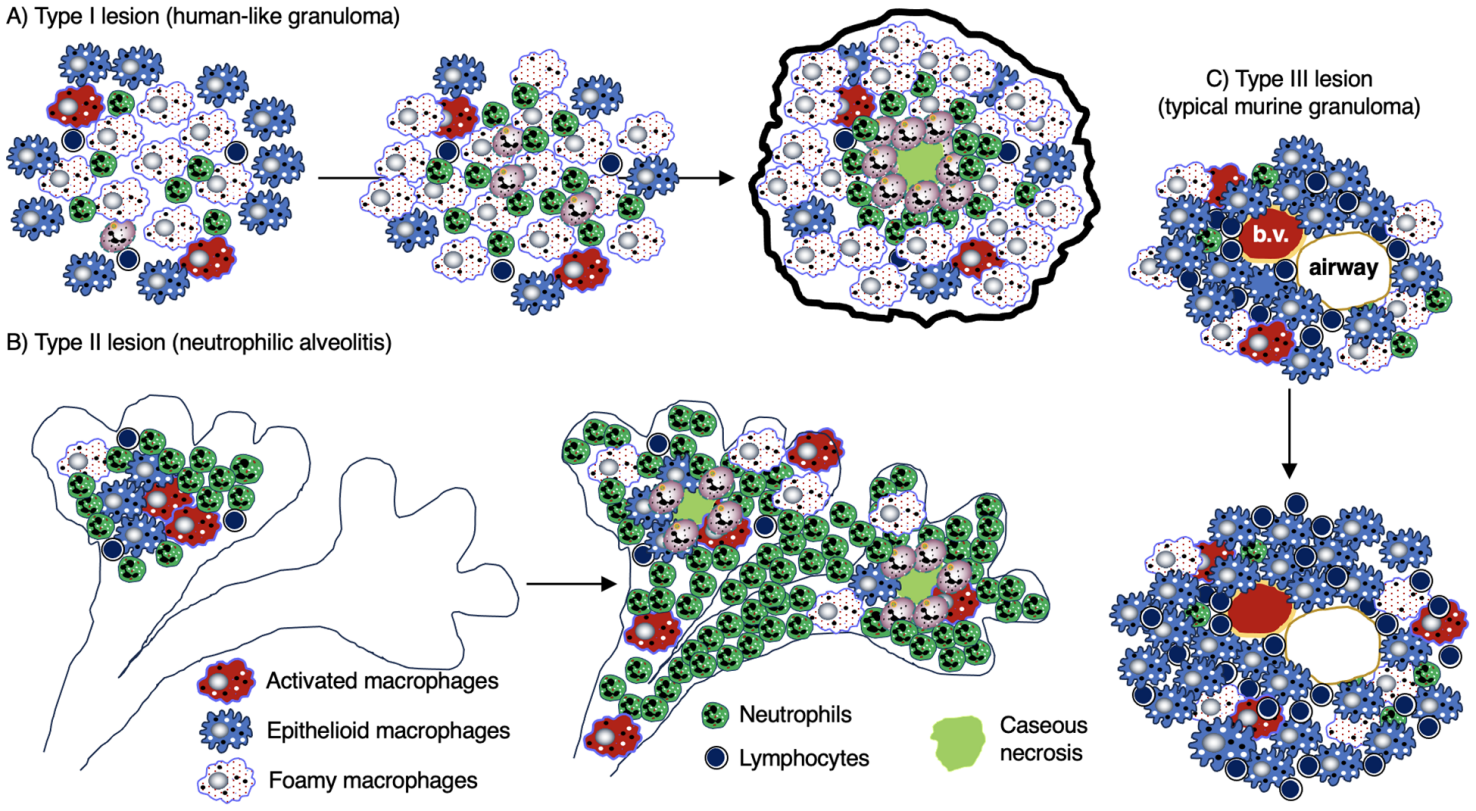
Different types of TB lung lesions in the C3HeB/FeJ mice. Three types of histopathologic TB lesions are seen in the C3HeB/FeJ mice. **(A)** Type 1 lesion is mainly comprised of foamy macrophages, few epithelioid macrophages and lymphocytes, and a caseous center surrounded by neutrophils. This lesion most resembles to that seen in human TB. **(B)** Type 2 lesion is the most severe form and is characterized by a neutrophilic, necrotizing alveolitis with occlusion of the terminal bronchioles and caseous necrosis. **(C)** Type 3 lesion is comprised mainly of infiltration of epithelioid macrophages and lymphocytes, smaller numbers of foamy and activated macrophages and neutrophils around the airway and blood vessel pair. This is the typical lesion seen in most other mouse strains. TB, tuberculosis.

While susceptibility to TB can be affected by the virulence of *Mtb* strain used, route of infection, and the inoculum dose (373), the genetic background of mice plays a critical role with *Mtb* infection. Many distinct mouse lineages exist with varying genetic backgrounds. Historically, these lineages are divided into two main categories: “resistant” or “susceptible” strains. Currently, the most common mouse lineages used in TB research are the BALB/c and C57BL/6 mice (“resistant” strains) and the C3HeB/FeJ, DBA/2, and 129/Sv mice (“susceptible” strains). The C3HeB/FeJ strain is regarded as a valuable resource for host-pathogen and drug response characterization since it better mimics histopathologic features of human TB: well-formed, hypoxic TB granulomas with central necrosis and liquefaction, elements not seen in other inbred strains (34, 326, 374, 375). Indeed, C3HeB/FeJ mice infected with *Mtb* may develop three morphologically distinct lesion types in the lungs (**Figures 11A–C**) (34). The unique susceptibility of C3HeB/FeJ mice to TB is due to *de novo* mutation and defective expression of the *Ipr1* gene located within the sst1 (super-susceptibility-to-tuberculosis 1) locus (376, 377). The *sst1* locus from C3HeB/FeJ has been engineered into a C57BL/6 background, termed congenic B6.C3H-*sst1* which harbored a 10–fold higher bacterial burden at four weeks post-infection and displayed large necrotic lesions in histopathology evaluations (376, 378). The B6.C3H-sst1 strain has helped demonstrate that the sst1 locus independently defines necrotic lesion progression in the mouse model (376).

The Collaborative Cross (CC) mice are a multi-parental mouse genetic reference population characterized by genetic polymorphism with a spectrum of sensitivity to *Mtb* infection (379–381). Bacterial burden after intravenous infection with *Mtb* ranges over 1000-fold across these strains (379). This CC strain catalog has advanced our understanding of the interplay of different genes on host susceptibility to TB by enabling the ability to reference and isolate key genetic loci influencing that sensitivity. For example, CC042 strains exhibit deficient IFNγ production which correlated with fewer macrophages and T cells being recruited to the lungs but enhanced neutrophil infiltration and necrotic lesions compared to C57BL/6 mice (380). In addition to informing which immune components provide host protection, the CC or diversity outbred (DO) mouse strains have also been shown to impact the effectiveness of TB vaccines and thus more likely to be more informative and a better alternative to inbred mouse strains. A DO model inherently lowers the power available to decipher key genetic components as each genotype is only present in a single animal, whereas the CC model retains diversity of the DO model while powering studies with repeatable and well powered “n” per genotype (382). Both CC and DO modeling better recapitulates the diversity and spectrum of TB disease observed in human clinical cases, including interrogation of pulmonary lesions and granuloma formation.

One notable disadvantage of the TB mouse model is that *Mtb* infection results in a slowly progressive infection that eventually leads to death, a model of primary progressive TB rather than post-primary TB or latent TB infection (323, 383). However, two models have been reported for latent TB in mice (with potential for reactivation): *(i)* the use of antibiotics to eliminate actively replicating *Mtb* but not sterilizing all *Mtb* (the Cornell model) (384) and *(ii)* intradermal (ear) inoculation of mice that produced a long-term clinical state characterized by the presence of *Mtb*-specific T cells but no overt signs of active TB (385). Major gaps in the field also include understanding subclinical TB disease and biological features that promote *Mtb* transmission, which remain outside the capacity of the present mouse models.

Other shortcomings of mice in TB research include: *(i)* their inability to form caseating granulomas and lung cavitation except in the C3HeB/FeJ mouse (386, 387) or as part of an experimental condition, such as mice that received intraperitoneal injection with TDM in oil prior to infection with *Mtb* (185, 295); *(ii)* predominance of loosely formed granulomatous lesion (Type III lesions mentioned in **Figure 11**), whereas humans form discrete granulomas (34, 266); *(iii)* absence of hypoxia in mouse granulomas (with the exception of the C3HeB/FeJ strain) and thus murine macrophages may be more likely to undergo apoptosis rather than necrosis (266, 388); *(iv)* localization of *Mtb* intracellularly in non-necrotic lesions in commonly used mouse strains (389), whereas in human and other animal models, *Mtb* primarily resides extracellularly (372, 387, 390); thus, the sterilizing activity of new TB drug/regimen seen in mice may not be observed in other animal models or human clinical trials (391); *(v)* alterations in how the CC mouse strains with genetic polymorphisms respond to TB vaccines (392); and *(vi)* differences in antigen clusters between mice and humans that may impact host immunity to *Mtb*; *e.g.*, mice do not possess a homolog of human IL-32, a cytokine associated with host protection in human macrophages and in a transgenic murine model that expresses human IL-32γ (393, 394). The clinical and pathologic features of *Mtb*-infected mice that mimic human pulmonary TB are described in **Table 4**.

### 6.3 Guinea pigs

Guinea pigs were one of the first experimental animals used to study TB. Some of the earliest studies using this model were conducted by Robert Koch and Max Lurie in the late 19^th^ and early 20^th^ centuries, respectively (395–397). Currently, the outbred Dunkin-Hartley strain is the most common strain used in research (398). Guinea pigs display many features during *Mtb* infection that are similar to humans: *(i)* newborn guinea pigs have mature lympho-myeloid system like that found in human infants; *(ii)* their hormonal and immunological phenotypes; *(iii)* guinea pigs also do not synthesize vitamin C but must obtain it from their diet; *(iv)* guineas pigs are glucocorticoid-resistant (324); and *(v)* presence of necrotic primary granuloma after low-dose *Mtb* infection (323–325).

Guinea pigs are also highly susceptible to *Mtb*, making them good models for primary progressive disease. They develop pulmonary TB with very low doses of *Mtb* (as low as 2-4 CFU per animal) (399) as well as with exposure to exhaust ambient air from human TB patients (400) or to neighboring *Mtb*-infected guinea pigs within the same room but in different cages (397). Lurie also showed that concomitant respiratory and enteric infection with *Mtb* of guinea pigs resulted in more chronic respiratory TB whereas only respiratory inoculation with *Mtb* resulted in more acute respiratory TB (396). Although it has been argued for many years that a latent TB model and thus post-primary (reactivation) TB are not possible in guinea pigs due to their increased susceptibility (325, 395, 401), some have found it possible to do so (402, 403). Because guinea pigs are also well protected by the BCG vaccine and respond well to anti-TB antibiotics, they are useful for drug and vaccine studies (399, 404–411).

Disadvantages of guinea pigs in TB research include: *(i)* greater expense than mice; *(ii)* most *Mtb* infections of guinea pigs model primary progressive disease than post-primary TB; *(iii)* more limited immunologic reagents than for mice. However, successful cloning of various cytokines (IFNγ, IL-4, IL-10, and IL-17) from guinea pigs expands the ability to characterize the immune response to *Mtb* (412–415). The clinical and pathologic features of *Mtb*-infected guinea pigs that mimic human pulmonary TB are described in **Table 4**.

### 6.4 Rabbits

Nearly 100 years ago, Lurie and colleagues developed a rabbit model of TB, wherein rabbits with varying levels of *Mtb* resistance were developed (416–418). Rabbits are useful to model both human latent TB infection and active TB, developing caseating granulomas that may liquify and cavitate (323, 395, 419–425). It has also been used to distinguish between protective cellular immunity and potentially damaging mechanisms of “delayed type hypersensitivity” (426–428).

The severity of rabbit TB lung pathology can be influenced by two main factors: the specific *Mtb* strain used; *e.g.*, Erdman *Mtb* was found to cause more severe pathology and worse outcomes than *Mtb* CDC1551 and H37Rv (424, 429) and the rabbit genotype; *e.g.*, inbred rabbits are more susceptible to *Mtb*, resulting in the formation of lung granulomas and cavities, than outbred rabbit strains (395, 424).

Another way that rabbits mimic human TB infection is that they show a spectrum of disease outcome, including the development of latent TB infection in the TB-resistant outbred strain of New Zealand white rabbits (424, 425, 430). Lung cavities are also common with serial low-dose *Mtb* infection of rabbits (431–433). Serial CT scans demonstrated that the cavities developed from areas of necrotizing pneumonia in the dorsal-caudal region of the rabbit lungs – anatomically analogous to the apical region of the human lung – pulmonary segments with the greatest mechanical stress, decreased blood flow, and highest partial pressure of alveolar O_2_ in the normal position of rabbits (311–313, 319, 431). Disadvantages of rabbits include larger housing requirements, greater expense, more difficulty in handling, greater challenge in gene manipulation, and lesser repertoire of immunologic reagents compared to mouse models (323, 434). The clinical and pathologic features of *Mtb*-infected rabbits that mimic human pulmonary TB are described in **Table 4**.

### 6.5 Mini pigs

Mini pigs have been used to model primary progressive TB (435, 436). Like guinea pigs, spread of *Mtb* may occur between infected mini pigs and their previously uninfected neighbors (436). The advantage of the mini pig model is that the anatomical structure of the lungs is like that seen in humans, with the normal presence of intralobular septae that become thickened following encapsulation of granulomas, which prevents the development of new lesions (435). This encapsulation – present in humans but not in rodents or NHP – makes the mini pigs a promising model to study latency because drainage of latent bacilli into the airways or alveolar spaces (435, 436). However, mini pigs also have the disadvantage of being expensive and large (up to 150 pounds). The clinical and pathologic features of *Mtb*-infected mini pigs that mimic human pulmonary TB are described in **Table 4**.

### 6.6 Goats

Goats are natural hosts of *M. caprae* and *M. bovis* and have been used to study bacillary load, pathology, and vaccine efficiency. Their high susceptibility and the fact that they mimic human infection by developing caseous necrotizing granulomas as well as cavities make them good models for studying TB (437). Compared to other experimental animals, goats are also more like humans in terms of size, body weight, and respiratory anatomy (266, 438). In goats, it was shown that the immune response varied depending on the route of BCG vaccination; *i.e.*, a negative intradermal or interferon-gamma release assay test for latent TB infection was more likely to be observed following oral administration of heat-inactivated *M. bovis* vaccine compared to the intramuscular route (439). Given that goats may develop TB lesions like that seen in humans and that either heat-inactivated *M. bovis* vaccine and BCG vaccine showed efficacy against *M. caprae* (440), goats are potential models to study vaccine efficacy. The clinical and pathologic features of *Mtb*-infected goats that mimic human pulmonary TB are described in **Table 4**.

### 6.7 Non-human primates

In early 1900’s, rhesus macaques were used in experimental TB by Dr. Gerald B. Webb and his colleagues at his home in Colorado Springs, Colorado (441, 442). Their work was involved subcutaneous injections of *Mtb* in the NHPs. Subsequent experimental TB work with rhesus macaques from ~1950-1970 also involved vaccination with intravenous BCG (443, 444). Since 1996, the Philippine cynomolgus macaque was utilized for experimental TB, showing that depending on the dose, either active TB or latent TB infection may develop (229, 445). Cynomolgus macaques primed with BCG and then boosted with the *Mtb72F/AS02A* vaccine showed long-term protection against TB (446). Whereas the control monkeys showed granulomas with caseous centers, extensive edema, and were fused, the granulomas of the NHP vaccinated sequentially with BCG and *Mtb*72F/AS02A were discrete lesions with no necrosis or edema (446). More recently, marmosets, which are much smaller than the macaques, have been used in experimental TB (447).

NHPs are likely the most accurate model in human TB research because of their similarities to humans genetically, immunologically (*e.g.*, functional MHC regions, T-cell antigen receptors, and other cellular features that are relevant for vaccine testing), clinically (manifesting a spectrum of TB from latent infection to active disease), pathologically (including pneumonia, various forms of granulomas, and cavitary disease) and pharmacokinetically (with relevance for testing new drugs) (229, 326, 448–457). Limitations of the NHP model are more logistical – smaller sample sizes and time points due to cost and more difficulty in handling (456). However, serial radiographic imaging of a relatively large animal – namely, chest radiograph, CT, magnetic resistance imaging, and ^18^FDG-PET-CT to detect areas of increased metabolic activity to indicate host immune response to the infection (458–460) – have been highly informative. The clinical and pathologic features of *Mtb*-infected NHP that mimic human pulmonary TB are described in **Table 4**. The key clinical, radiographic, and pathological differences between the rhesus and cynomolgus macaques relevant for TB are shown in **Table 4**.

## 7 Summary and future perspective

Substantial progress has been made in our understanding of the host immune responses that either protect against TB or contribute to TB pathogenesis. Much of the work have focused on the granulomas and the molecular and cellular components that participate in their formation in experimental animals as well as re-analysis of archived and more contemporaneous human TB tissues. While descriptive analyses of the different cell types in the granuloma architecture have been dominating, the role of soluble mediators (cytokines, chemokines, growth factors) and metabolic studies (hypoxia, aerobic glycolysis, and activity of various cellular enzymes such as MMPs, IDO-1, and HO-1) have enriched our understanding of TB granulomas but have also raised new avenues of research. One emerging paradigm is that either a deficiency or excess of certain host-derived molecular and cellular components may be harmful to the host which, in turn, allow for the proliferation and persistence of *Mtb*. While new imaging techniques such as MIBI-TOF (multiplexed ion beam imaging by time of flight) and PET-CT using the radioactive tracers deoxy-2-[^18^F]-fluoro-D-glucose and [^18^F]- fluoromisonidazole have contributed to our understanding of TB, it is important to understand their limitations. For example, one overarching limitation is our inability to analyze immunological and microbiological data simultaneously in individual granulomatous TB lesions *in vivo* over time. Rather, “snapshots” of events limits our ability to interpret whether the myriad of molecular and cellular components that can be found in active TB are: *(i)* the main drivers of TB pathogenesis (causing loss of host protection), *(ii)* attempts to correct an inadequate initial immune response, or *(iii)* possibly both depending on the temporality of the responses. While some of these limitations can be overcome by targeted genetic disruption of specific molecular or genetic components, inherent limitations of these models to human TB remain. A better understanding of the immune correlates of protection may also help guide the development of antimicrobial agents that can target privileged sites of *Mtb* and of host-directed therapies. While “high-tech” approaches to better understand granulomas and other TB lesions are occurring, we ought not lose sight of the value of “low-tech” foundational principles of anatomy, physiology, and chemistry that should be included in both the experimental studies and care of TB patients.
